# A Combined Experimental and Computational Study of Halogen and Hydrogen Bonding in Molecular Salts of 5-Bromocytosine

**DOI:** 10.3390/molecules26113111

**Published:** 2021-05-23

**Authors:** Massimiliano Aschi, Giorgia Toto Brocchi, Gustavo Portalone

**Affiliations:** 1 Dipartimento di Scienze Fisiche e Chimiche, Università degli Studi di L’Aquila, Via Vetoio 10, 67100 Coppito, Italy; massimiliano.aschi@univaq.it; 2 Dipartimento di Chimica, Università di Roma “La Sapienza”, Piazzale Aldo Moro 5, 00185 Roma, Italy; Totobrocchi.1699396@studenti.uniroma1.it

**Keywords:** 5-bromocytosine, modified nucleobases, DNA, biomolecular engineering, molecular recognition, halogen bonding, hydrogen bonding, X-ray diffraction, DFT, QTAIM

## Abstract

Although natural or artificial modified pyrimidine nucleobases represent important molecules with valuable properties as constituents of DNA and RNA, no systematic analyses of the structural aspects of bromo derivatives of cytosine have appeared so far in the literature. In view of the biochemical and pharmaceutical relevance of these compounds, six different crystals containing proton-transfer derivatives of 5-bromocytosine are prepared and analyzed in the solid-state by single crystal X-ray diffraction. All six compounds are organic salts, with proton transfer occurring to the N_imino_ atom of the pyridine ring. Experimental results are then complemented with Hirshfeld surface analysis to quantitively evaluate the contribution of different intermolecular interactions in the crystal packing. Furthermore, theoretical calculations, based on different arrangements of molecules extracted from the crystal structure determinations, are carried out to analyze the formation mechanism of halogen bonds (XBs) in these compounds and provide insights into the nature and strength of the observed interactions. The results show that the supramolecular architectures of the six molecular salts involve extensive classical intermolecular hydrogen bonds. However, in all but one proton-transfer adducts, weak to moderate XBs are revealed by C–Br^…^O short contacts between the bromine atom in the fifth position, which acts as XB donor (electron acceptor). Moreover, the lone pair electrons of the oxygen atom of adjacent pyrimidine nucleobases and/or counterions or water molecules, which acts as XB acceptor (electron donor).

## 1. Introduction

Natural or chemical modified nucleobases represent an important class of molecules with considerable properties as constituents of DNA and RNA. These modified nucleobases are profitable to originate new nucleic acid structures, to identify binding sites, and to broaden the suitability of nucleic acids in the field of diagnostics, therapeutics, biomolecular engineering, and nanotechnology [1,2]. Consequently, it is not surprising that in the past couple of decades, considerable attention has been attracted by the role played by noncovalent interactions flanking hydrogen bond (HB) and engaging halogen atoms in the molecular recognition and supramolecular assembly of *halo* (*halogen*)-modified nucleobases. As a result, although in these systems HBs remain prevalent among other coexisting noncovalent interactions, the significance of halogen bonds (XBs) today is largely recognized [3,4,5,6,7].

In halogen-bonded complexes, the halogen atom in one molecule can interact favorably with the negative site of an adjacent molecule, due to an attraction between the positively charged site of a covalently bound halogen atom (σ-hole) and an electron donor species, usually a neutral or ionic Lewis base [8]. However, as the strength of an XB depends on the size and polarizability of the halogen atom, which both increase with the halogen atomic number, F atom rarely forms halogen bonds, and typically Cl, Br, and I atoms are considered in biologically relevant compounds [5].

In DNA polymers, high-resolution structures of complexes with halogenated ligands are uncommon. Nonetheless, among DNA systems showing halo-substituted pyrimidine nucleobases, the structural aspects of XBs have been inferred from experimental and theoretical studies, and bromine and oxygen atoms appear to be the optimal electron acceptors and donors, respectively [5].

As concerns halouracils as constituents of nucleic acids, from a survey in the Protein Data Bank (PDB) [9], crystallographic evidence indicates that 5-bromouracil stabilizes a Holliday junction, a branched DNA structure containing four double-stranded arms joined together, through the formation of a short Br···O intermolecular contact, ≈3.00 Å. This contact corresponds to an *R*_XB_* (*R*_XB_ is defined as *R*_XB_ = *d*_XB_/*d*_max_ [10], where *d*_XB_ [Å] is the distance between the donor *X* and the acceptor atoms *B*, and *d*_max_ is the sum of the vdW radii [Å] for the *X* and *B* interacting atoms [11]) equal to 0.89. Remarkably, nucleic acid folding is sensitive to the exact position (C5) where the halogenation occurs in the pyrimidine nucleobase [12]. This result agrees with theoretical studies on a halogenated DNA fragment, based on the stacked-X form in the sequence d(CCAGTACBr^5^UGG), which show that nucleotide C–Br^…^O–P contact linking 5-bromouracil to an adjacent phosphate oxygen influences the local conformation of the model system and contributes to stabilizing its flexible backbone [13]. Very recently, a comprehensive PDB survey revealed that also moderately strong intramolecular XBs between bromine atoms of 5-bromouracil and oxygen atoms of the backbone phosphate groups can enhance the stability of DNA and RNA molecules [14].

Regarding halo-modified cytosines in nucleic acids, 5-bromoderivatives deserve special attention in epigenetic control of gene expression. Bromine atom can mimic the behavior of a methyl group in DNA-protein interactions, and 5-bromocytosine can result in aberrant methylation of cytosine during replication of DNA, as methyl-binding proteins cannot discriminate methylated from halogenated DNA [15,16]. Moreover, 5-bromocytosine can be incorporated into cellular DNA to facilitate DNA-strand breaks and crosslinks upon UV irradiation, and has been proposed for clinical use as a photosensitizer [17]. Due to the capability of this halocytosine to form supramolecular assemblies through XBs and HBs, it has been shown that the stacking pattern, which allows close contacts between the halogen and the oxygen atoms of two adjacent bases, enhances the stability of hairpin triples DNAs containing 5-bromocytosine [18,19].

It is also well recognized that nucleobases (and halonucleobases) can be protonated at physiological pH, and the protonation affects the biological functions of nucleic acids, as a consequence of changes in the hydrogen-bonding characteristics of base pairing. Numerous studies, based on the investigation of crystal and solution structures, demonstrate that protonated nucleobases are implicated in RNA catalysis and control folding and stability of RNA and DNA, due to their capacity to form specific noncovalent intermolecular interactions [20,21]. A remarkable example of structural versatility due to the nucleobase protonation is offered by the intercalated motif (*i*-motif) in DNA. In the *i*-motif, based on the association of cytosine molecules protonated under neutral or slightly acidic conditions to form neutral cytosine—hemi-protonated cytosine base pairs (C:CH^+^), two parallel-stranded duplexes are held together in an antiparallel orientation by intercalated C:CH^+^ pairs [22,23,24,25]. Noteworthy, by decreasing the proton affinity of the N3 atom, 5-halogenation of 1-methylcytosine does not destroy the *i*-motif in DNA, but may alter the number of trinucleotide repeats required to induce the structural conversion from Watson and Crick (*WC*) base-pairing to the *i*-motif association [26].

Surprisingly, in spite of its potential interest, little is known about the structural aspects of 5-bromocytosine and its protonated derivative in the solid state. A search in the Cambridge Crystallographic Database (CSD version 5.42, February 2021 update) [27] for neutral and protonated unsubstituted 5-bromocytosine gave five hits. From this survey, three hits contained neutral 5-bromocytosine (refcodes: BCYTGA, BRCPDG, and BRCYTS [28,29,30]), and only two hits contained N_imino_(N3)-protonated 5-bromocytosine, (refcodes: HABKEA and NUZKEY [31,32]). Adopting a cutoff value of 0.96 for the interaction ratio *R*_XB_, three structures (BCYTGA, BRCPDG, and HABKEA) exhibited weak almost linear XBs (C–X^…^B bond angle ≥ 155°) in which bromine atoms act as XB donors, and the strongest XB interaction was found in one of the two structures containing the 5-bromocytosinium cation (HABKEA, *R*_XB_ = 0.88).

In the light of the above observations, we believed important to conduct a systematic structure analysis for molecular salts involving N_imino_-protonated 5-bromocytosine and simple counterions, which can both act as hydrogen and/or halogen bond donor/acceptor. In particular, the present study aims to determine qualitatively and quantitatively the interactions of protonated 5-bromocytosines with themselves, counterions, and water molecules by a combined experimental and computational approach.

**Scheme 1:**
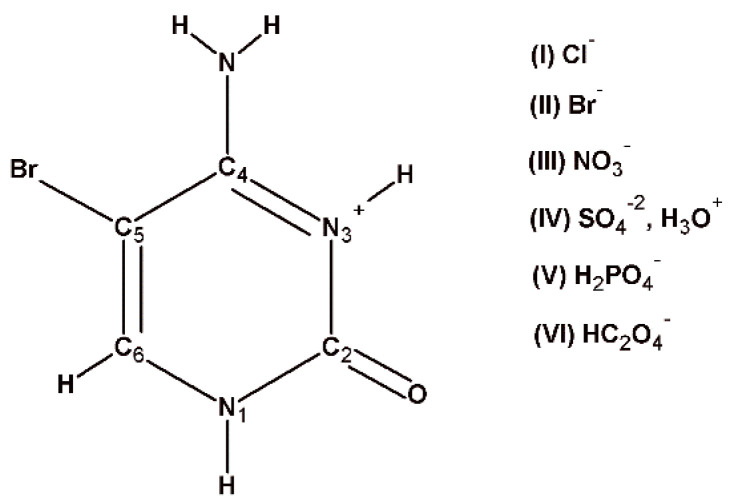
Chemical components of the compounds (**I**–**VI**) investigated in this study.

For this purpose, we prepared and crystallized six different molecular salts containing proton-transfer derivatives of 5-bromocytosine (Scheme 1).

5-bromocytosine, as cytosine [33], can exist in different tautomeric forms, which can induce modifications in the normal base pairing. Although the possibility of tautomerization of 5-bromocytosine has been calculated to be much less likely than that of cytosine, analyses of FTIR and Raman spectra of this halonucleobase, supported by ab initio and DFT calculations, demonstrate the prevalence in the gas phase of the aromatic amino-2-hydroxy *cis* tautomer among the nine possible tautomeric forms originated by proton migration involving the O, N1, N3, and N_amino_ atoms. On the contrary, by using a tetrameric fragment of 5-bromocytosine as a simplified model of the crystal unit cell, only the simulated spectra of tetramer nonaromatic 1*H*-amino-oxo forms satisfactorily reproduce the experimental spectra in the solid state [34]. These results confirm the preference for only one tautomeric form in the crystal of 5-bromocytosine, i.e., the tautomer, which is the most frequently present in nucleic acids [35].

Due to the halo substitution at the fifth position, 5-bromocytosine, as a base, is moderately stronger than cytosine (*p*K_a1_ = 3.04 and 4.61, respectively [36]), and in the presence of acids, can be easily protonated at the N3 atom. In the solid state, the existence of neutral or charged molecular adducts formed through proton transfer reactions can be foreseen by the Δ*p*K_a_ value (*p*K_a_ (conjugate acid of the base)—*p*K_a_ (acid), *p*K_a_’s are for an aqueous solution at 25 °C). For Δ*p*K_a_ > 3, salts of the type B^+^–H^...^A^−^ are expected, whereas Δ*p*K_a_ < 0 will almost exclusively result in neutral component B^...^H–A cocrystal. Nevertheless, as in the case of molecular salts (**V**) and (**VI**) (Δ*p*K_a_ = 3.04 − 1.96 = 1.08 and 3.04 − 1.27 = 1.77, respectively), when 0 < Δ*p*K_a_ < 3 the pH dependence to control crystal formation seems inappropriate and overlap of the cocrystal to the salt continuum is observed [37]. It should be noted that in a crystal, due to the anisotropic molecular environment, polarization via strong HBs can play a key role in determining the formation of a salt or a cocrystal. Therefore, as HBs may be considered the partially activated precursors to proton-transfer reactions [38], to predict the salt or cocrystal formation, additional factors, other than Δ*p*K_a_, such as dynamic proton disorder or temperature dependency of proton transfer, should be considered [39]. Recently, the dependency of proton-transfer reactions on temperature has been investigated in molecular systems showing short, strong hydrogen bonds, i.e., HBs shorter than 2.55 Å, (SSHBs) [40]. To this purpose, the crystal structures of eight molecular complexes, exhibiting SSHBs and containing *N*,*N*-dimethylurea or isonicotinamide coupled with organic acid coformers, have been determined by multitemperature single crystal synchrotron X-ray diffraction in the range 100–350 K. Noteworthy, two of the eight complexes examined, (1:1) *N*,*N*-dimethylurea/2,4-dinitrobenzoic acid and (2:1) isonicotinamide/phthalic acid, showed temperature dependency of proton-transfer processes across HBs. Indeed, in both of them, transition occurs between the salt (100 K) and the cocrystal (300–350 K), and an almost complete proton migration to the acidic coformer was found in the neutral adducts.

The supramolecular assembly of the six molecular salts was, thus, investigated in the solid state through single crystal X-ray diffraction. Two different computational approaches were then employed: Hirshfeld surface and decomposed 2D-fingerprint plots, to quantitively evaluate the contribution of different intermolecular interactions in the crystal packing [41]; Bader’s quantum theory of atoms-in-molecules (QTAIM) [42], to provide a more quantitative estimation of the energies and some of the topological features of supramolecular contacts which involve hydrogen and halogen atoms in the formation of the complex environment of such biochemical models.

This article considers a continuation of our long interest in the design and construction of supramolecular architectures of neutral and protonated pyrimidine nucleobases [43,44,45,46,47,48,49,50,51,52,53] and in experimental/computational analyses of simple biological systems exhibiting halogen bonding via alternative donors, i.e., halogen-bond donors not boosted by fluorination of the skeletal structure to which the halogen-bond donors are attached [10,54,55].

## 2. Results and Discussion

### 2.1. Structural Analysis

In this section, we report the crystal structure analysis for all the investigated systems accompanied by the computational QTAIMS analysis aimed at quantitatively describe the localized HBs and XBs. To this purpose, we have first investigated the actual existence of bond critical points (rank 3 and signature −1), hereafter simply termed as CPs, in correspondence to the pair interactions that emerged from the crystallographic structural analysis. Subsequently, for each CP, we have evaluated the topological and energetic features following the criterion by Espinosa et al. [56,57]. For the halogen bond interactions, the energy was estimated using a slightly different approach proposed by Tsirelson [58]. The balance between the Lagrangian kinetic energy G(**r**) and the potential energy density V(**r**) at the CP were then utilized for quantifying the noncovalent character (−G/V > 1) of all the corresponding pair interactions.

**Figure 1:**
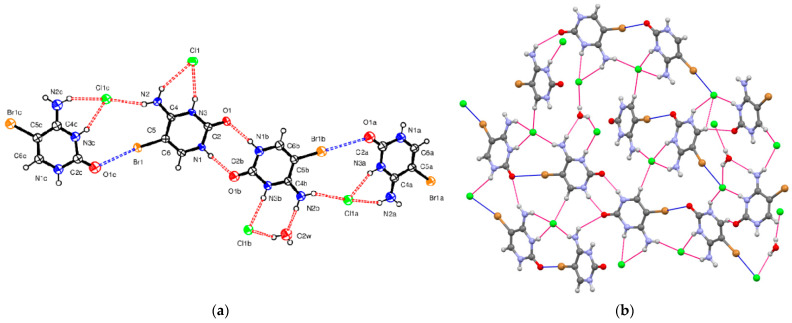
(**a**) The asymmetric unit of compound (**I**), showing the adopted atom-numbering scheme. Displacement ellipsoids are at the 50% probability level. H atoms are shown as small spheres of arbitrary radii. Hydrogen bonding is shown as red dashed lines. Halogen bonding is shown as blue dashed lines. (**b**) Part of the crystal structure of (**I**). All atoms are shown as small spheres of arbitrary radii. Hydrogen bonding is shown as red dashed lines. Halogen bonding is shown as blue dashed lines.

5-Bromocytosinium chloride, (**I**), crystallizes in the orthorhombic space group Pbca in the cation/anion ratio 1:1, but the asymmetric unit consists of four 1*H*,3*H*-5-bromocytosinium cations protonated at the N3 atom, four chloride anions and one water molecule of crystallization joined together to form one-dimensional multicomponent planar aggregate (Figure 1a). The occurrence of more than one molecule in the asymmetric unit of ionic compounds is rather unusual [59,60], but a similar situation for N_imino_-protonated 5-bromocytosines have been encountered in the crystal structure of NUZKEY, which crystallizes with three 5-bromocytosinium cations, a hexachloro-platinum(IV) anion, one chloride anion and a water molecule in the asymmetric unit.

In the crystal packing of (**I**), shown in Figure 1b, in the planar aggregates, a pair of 5-bromocytosinium cations are linked together by N–H···O hydrogen bonds from the CONH pyrimidinium groups forming a cyclic hydrogen-bonded ring motif represented by graph-set notation R^2^_2_(8) [61]. These ion pairs are further connected into a zigzag chain via N–H^…^ Cl^−^ hydrogen bonds and two almost linear C–Br^…^O contacts (3.03 Å, 165°, and 3.26 Å, 175°) involving the carbonyl oxygen atom of adjacent peripheral 5-bromocytosinium cations, to give 1D chains. The XB ratios *R*_XB_ vary between 0.90 and 0.96, indicating weak interaction strength. In this respect, the XB interaction energies (between 1.5 and 2.0 kcal/mol) and −G(**r**)/V(**r**) ratio (between 1.2 and 1.3), evaluated based on topological analysis on the corresponding CPs, clearly confirm the low, but not negligible strength of these interactions, as well as their genuine noncovalent character. These chains are then united by N–H^…^Cl^−^, N^+^–H^…^Cl^−^, N–H^…^O_w_ and O_w_–H^…^Cl^−^ hydrogen bonds and two C–Br^…^Cl^−^ contacts involving Cl^−^ ions (3.07 Å, 171°, *R*_XB_ = 0.86 and 3.22 Å, 165°, *R*_XB_ = 0.90, respectively) into sheets. In correspondence with the latter interactions, CPs have been localized by the topological analysis with an estimated interaction energy of 1.2 kcal/mol. No relevant intermolecular HBs involving the Br atom were observed (Table 1). From the topological analysis, all the HBs turn out to be characterized by −G(**r**)/V(**r**) close to unity with interaction energies varying from 3.0 to 10.0 kcal/mol. In particular, rather strong interactions are found for all the N^+^–H^…^Cl^−^, and although to a lesser extent, even N–H^…^Cl^−^ HBs. Rather surprisingly, our calculations have revealed significantly E_int_ (Internal Energy) values also for the HBs involving uncharged N1–H1^…^O1B and N1B–H1B^…^O1. The latter result can be partially rationalized based on the relatively high value of the Laplacian of the density observed for the corresponding pair interactions.

**Figure 2:**
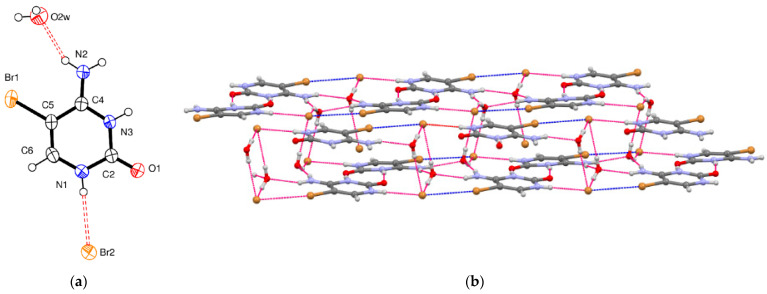
(**a**) The asymmetric unit of compound (**II**), showing the adopted atom-numbering scheme. Displacement ellipsoids are at the 50% probability level. H atoms are shown as small spheres of arbitrary radii. Hydrogen bonding is shown as red dashed lines. (**b**) Part of the crystal structure of (**II**). All atoms are shown as small spheres of arbitrary radii. Hydrogen bonding is shown as red dashed lines. Halogen bonding is shown as blue dashed lines.

5-Bromocytosinium bromide, (**II**), crystallizes in the monoclinic space group *C*2/*c*, and the asymmetric unit consists of one 1*H*,3*H*-5-bromocytosinium cation protonated at the N3 atom, one bromide anion, and one water molecule of crystallization (Figure 2a).

The packing of (**II**) is shown in Figure 2b. Pairs of 5-bromocytosinium cations are linked together by equivalent N^+^–H···O hydrogen bonds, which are significantly shorter than normal N–H^…^O intermolecular interactions (Table 2). These pairs then assemble in layers held together by N–H^…^O_w_ of R^2^_4_(8) graph motif involving the amino group of one protonated nucleobase and a nearby oxygen atom of a water molecule, and by N–H^…^Br^−^ and O_w_–H^…^Br^−^ hydrogen bonds to reconstruct the 3D network. All the HBs found in the crystal are confirmed by the topological analyses, which also indicates interaction energies in the range 3.0–9.0 kcal/mol. C–Br^…^O interactions do not emerge from the crystal structure analysis nor the topological analysis. On the other hand, weak (*R*_XB_ = 0.96) and almost linear C–Br^…^Br^−^ contacts (3.52 Å, 160°) involving Br^−^ counterions are present in the crystal and are also localized and characterized by topological analysis as relatively weak (E_int_ = 1.5 kcal/mol) and markedly noncovalent interactions (−G(r)/V(r) > 1). Moreover, in this case, as expected, the strongest interactions turn out to be in correspondence with the HBs involving the N^+^–H moiety.

**Figure 3:**
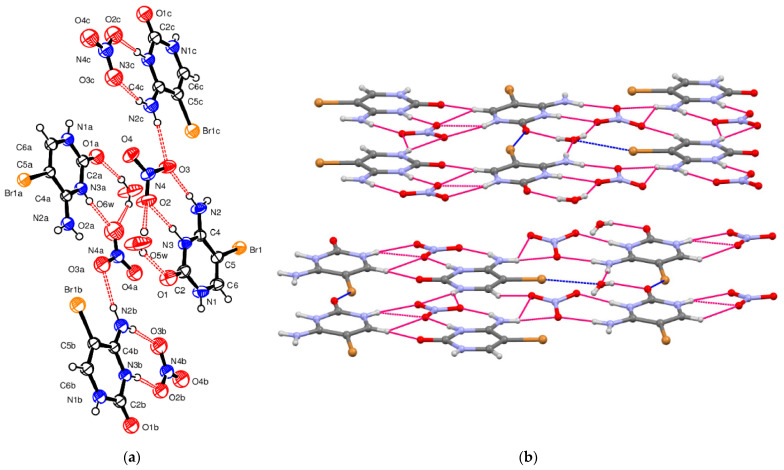
(**a**) The asymmetric unit of compound (**III**), showing the adopted atom-numbering scheme. Displacement ellipsoids are at the 50% probability level. H atoms are shown as small spheres of arbitrary radii. Hydrogen bonding is shown as red dashed lines. (**b**) Part of the crystal structure of (**III**). All atoms are shown as small spheres of arbitrary radii. Hydrogen bonding is shown as red dashed lines. Halogen bonding is shown as blue dashed lines.

5-Bromocytosinium nitrate, (**III**), crystallizes in the triclinic space group *P*-1 in the cation/anion ratio 1:1. As already mentioned above, for compound (**I**) and NUZKEY, multiple molecules are present in the asymmetric unit. Four 1*H*,3*H*-5-bromocytosinium cations protonated at the N3 atom, four nitrate anions, and two water molecules of crystallization are joined together to form a multicomponent aggregate (Figure 3a).

The packing of compound (**III**) is shown in Figure 3b. The ions assemble in layers through N–H^…^O^−^ and N^+^–HO^−^ hydrogen bonds (Table 3). For all of these HBs, the topological analysis has allowed us to locate as many CPs. The corresponding values of −G(r)/V(r) and E_int_, falling in the range 0.9–1.2 and 3.0–8.0 kcal/mol, respectively, describe these pair interactions as genuine noncovalent and rather stable HBs. As expected, the highest E_int_ values are found in correspondence with the N^+^–H^…^O interactions. There are two kinds of C–Br^…^O interactions (see also Table S6 in the Supplementary Information). The first one involves two carbonyl oxygen atoms (3.05 Å, 157°, *R*_XB_ = 0.90 and 3.16 Å, 150°, *R*_XB_ = 0.94, respectively), and according to QTAIMS analysis, is characterized by a rather high –G(**r**)/V(**r**) values (1.20 and 1.22) and by an appreciable interaction energy (3.6 and 2.4 kcal/mol). The second kind of C–Br^…^O interactions involves two oxygen atoms of water molecules (2.97 Å, 164°, *R*_XB_ = 0.76 and 3.03 Å, 161°, *R*_XB_ = 0.78, respectively), and similarly to the previous case, has revealed an appreciable interaction energy of 2.9 and 2.1 kcal/mol, respectively. No relevant intermolecular HBs involving the Br atom were observed. On the other hand, topological analysis has also located (see Table S7 in the Supplementary Information) quantitatively relevant noncovalent interlayers interactions (−G(**r**)/V(**r**) close to 1.0) involving N4, O2, and O3 atoms of nitrate anions with a relatively high E_int_ (between 7.0 and 8.0 kcal/mol).

**Figure 4:**
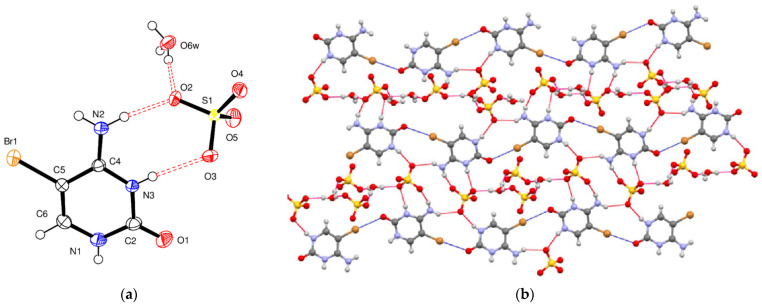
(**a**) The asymmetric unit of compound (**IV**), showing the adopted atom-numbering scheme. Displacement ellipsoids are at the 50% probability level. H atoms are shown as small spheres of arbitrary radii. Hydrogen bonding is shown as red dashed lines. (**b**) Part of the crystal structure of (**IV**). All atoms are shown as small spheres of arbitrary radii. Hydrogen bonding is shown as red dashed lines. Halogen bonding is shown as blue dashed lines.

5-Bromocytosinium sulfate, (**IV**), crystallizes in the monoclinic space group *P*2_1_/*c*, and the asymmetric unit consists of one 1*H*,3*H*-5-bromocytosinium cation protonated at the N3 atom, one sulfate anion, and one protonated water molecule of crystallization (Figure 4a). Short S–O bonds (1.458–1.483 (1) Å) in the slightly distorted tetrahedral sulfate anion indicate the absence of H atoms and delocalization of negative charge between them.

In the crystal structure of compound (**IV**), the ionic components are joined together essentially by two kinds of short-contact interactions. The first one corresponds to N–H^…^O^−^ hydrogen bonds involving the oxygen atoms of the sulfate anion. These interactions were also localized by QTAIMS analysis and characterized by interaction energy between 5.0 and 7.0 kcal/mol. The second type of interaction corresponds to an almost linear (2.96 Å, 175°) and rather weak (*R*_XB_ = 0.88) C–Br^…^O halogen bond involving bromine and carbonyl oxygen atoms of two adjacent 5-bromocytosinium cations, to give 1D zigzag chains (Figure 4b). The latter interaction has been indeed described by the topological analysis, and results in an interaction energy of 2.1 kcal/mol and a G(r)/V(r) value slightly larger than 1.20. These chains are then connected by N–H^…^O^−^ and N^+^–H^…^O^−^ hydrogen bonds with 1D chains formed by alternating sulfate and hydronium ions linked by two very strong O_w_–H^…^O^−^ interactions (Table 4) to form 2D layers. A short contact, C6–H6^…^O_5_ [3.169 (2) Å] has been confirmed by topological analysis showing a relatively low E_int_ (1.7 kcal/mol). The 2D layers, thus, formed are themselves linked into a three-dimensional network by the remaining very strong O_w_–H^…^O^−^ hydrogen bond. More precisely, the analysis of this part of the structure reveals a peculiar interaction characterized by an H_3_O^+^ moiety coordinating three sulfate anions, as also described in Table S10 in the Supporting Information, with strong HBs contacts whose interaction energies parallel the −G(**r**)/V(**r**) values of 0.90, 0.59 and 0.69. No relevant intermolecular HBs involving the Br atom were observed.

**Figure 5:**
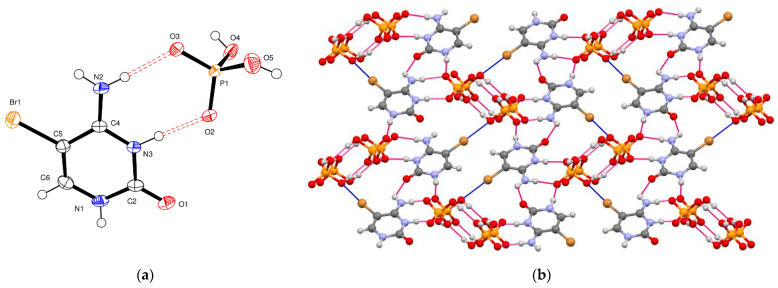
(**a**) The asymmetric unit of compound (**V**), showing the adopted atom-numbering scheme. Displacement ellipsoids are at the 50% probability level. H atoms are shown as small spheres of arbitrary radii. Hydrogen bonding is shown as red dashed lines. (**b**) Part of the crystal structure of (**V**). All atoms are shown as small spheres of arbitrary radii. Hydrogen bonding is shown as red dashed lines. Halogen bonding is shown as blue dashed lines.

5-Bromocytosinium dihydrogen monophosphate, (**V**), crystallizes in the monoclinic space group *P*2_1_/*c*, and the asymmetric unit contains one 1*H*,3*H*-5-bromocytosinium cation protonated at the N3 atom and one dihydrogen monophosphate anion (Figure 5a).

The single deprotonation of the phosphoric acid is reflected in the bond distances involving the P1–O bonds in the slightly distorted tetrahedral phosphate group. There are two short P1–O bonds of 1.493 (2) and 1.503 (2) Å to terminal atoms O2 and O3, respectively, and two longer bonds of 1.564 (2) and 1.569 (2) Å to atoms O4 and O5, bearing H atoms. These values correlate well with those reported for dihydrogen monophosphate anion in the crystal structure of 6-methylisocytosinium dihydrogen monophosphate adduct [51]. The similarity of P1–O2 and P1–O3 bond distances suggests delocalization of negative charge between the two bonds.

The supramolecular structure of compound (**V**) is based on a vast three-dimensional hydrogen-bonded network, which includes a combination of rather strong O–H^…^O and N–H^…^O hydrogen bonds (Figure 5b), as confirmed by the topological analysis. The hydrogen-bonding scheme involves all available donor/acceptor sites, but carbonyl O1, which remains partially unsaturated (Table 5). Within the cation chains, adjacent 5-bromocytosinium ions are linked head-to-tail through N–H^…^O hydrogen bonds, so forming infinite chains. Each dihydrogen monophosphate ion participates in five N–H^…^O and O–H^…^O hydrogen bonds with neighboring cations and anions as acceptor and in two O–H^…^O hydrogen bonds with neighboring anions as a donor. Through these interactions, dihydrogen monophosphates form pairs, all showing a rather high E_int_, of infinite chains cross-linked to cation chains. An approximately linear (3.04 Å, 171°) and rather weak (*R*_XB_ = 0.90) C–Br^…^O halogen bond involving the O5 oxygen atom connects pairs of 5-bromocytosinium and dihydrogen monophosphate ions. Similar XB interactions (3.00–3.01 Å, 172–173°) have been observed in the crystal structures of U2AF^65^-RNA complexes (U2AF = U2 auxiliary protein factor), where 5-bromouracil units are implicated in intramolecular XBs between Br atoms and O atoms belonging to nearby phosphate anions [62,63]. Topological analysis carried out on the latter contact has revealed interaction energy of 2.2 kcal/mol and a genuinely noncovalent character (−G(r)/V(r) > 1.2).

**Figure 6:**
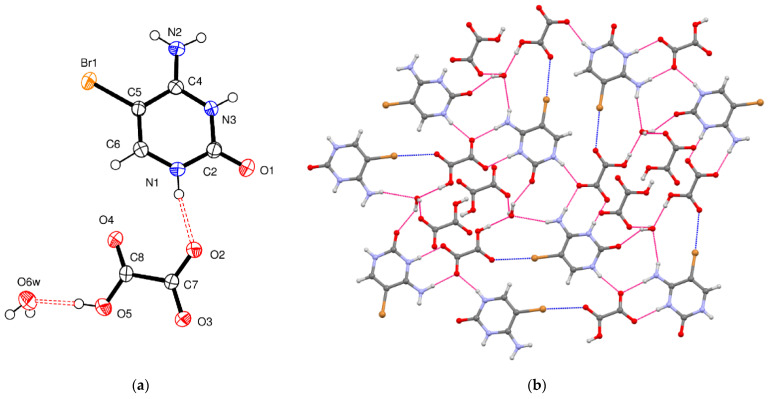
(**a**) The asymmetric unit of compound (**VI**), showing the adopted atom-numbering scheme. Displacement ellipsoids are at the 50% probability level. H atoms are shown as small spheres of arbitrary radii. Hydrogen bonding is shown as red dashed lines. (**b**) Part of the crystal structure of (**VI**). All atoms are shown as small spheres of arbitrary radii. Hydrogen bonding is shown as red dashed lines. Halogen bonding is shown as blue dashed lines.

5-Bromocytosinium hydrogen oxalate, (**VI**), crystallizes in the monoclinic space group *P*2_1_/*c*, and the asymmetric unit consists of one 1*H*,3*H*-5-bromocytosinium cation protonated at the N3 atom, one hydrogen oxalate anion, and one water molecule of crystallization (Figure 6a). The semi-oxalate anion is almost planar, as the dihedral angle formed by the planes defined by the CO_2_H and CO_2_^−^ non-H atoms is 5.2 (4)°. Bond distances around atom C7 [1.248–1.250 (3) Å] are typical of a carboxylate group, and the differences between them of 0.002 (3) Å indicates delocalization of the negative charge between the two O atoms. Bond distances around atom C8 (1.209(4) and 1.300 (4) Å) are consistent with the presence of a carboxylic acid group.

The crystal structure of compound (**VI**) is dominated by different structurally significant hydrogen bonds, formed by neutral and charged N–H^…^O and O–H^…^O interactions (Figure 6b). Two semi-oxalate anions are engaged in hydrogen bonds with two 5-bromocytosinium cations and two water molecules, forming an R^6^_6_(22) hydrogen-bonded ring. This subunit is then linked into a two-dimensional network by multiple hydrogen bonds, whose E_int_ is relatively strong by QTAIMS analysis, and a weak C–Br^…^O halogen bond (2.97 Å, 166°, *R*_XB_ = 0.88) between 5-bromocytosinium cations and hydrogen oxalate anions. This latter interaction is characterized by a relatively high −G(r)/V(r) value (1.20) and a value on E_int_ of 2.1 kcal/mol. As previously mentioned, water molecules play an important role in the cohesion of the crystal structure, as they maximize HB interactions with all potential donors and acceptors utilized. They form one N–H^…^O_w_ and one O–H^…^O_w_ hydrogen bonds connecting one 5-bromocytosinium cation and one semi-oxalate anion as donors, and two O_w_–H^…^O hydrogen bonds connecting one 5-bromocytosinium cation and one semi-oxalate anion as acceptors (Table 6). No relevant intermolecular HBs involving the Br atom were observed.

### 2.2. Full Interaction Maps Analysis

**Figure 7:**
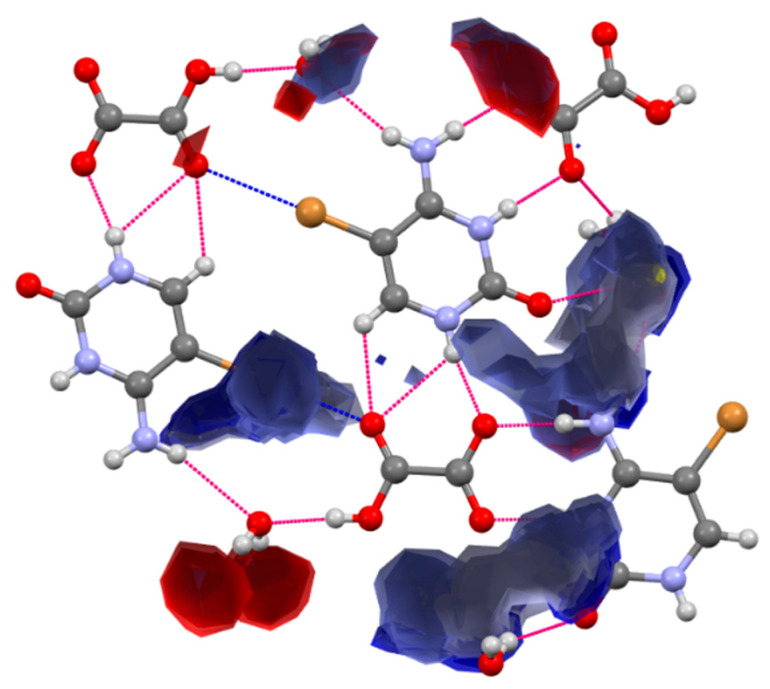
Visualization of the full interaction map calculated around the content of the asymmetric unit for compound (**VI**). Region of HB acceptor and donor likelihood are shown in red and blue, respectively. Acceptor peaks for halogen bonds are shown in violet. Hydrogen bonding is shown as red dashed lines. Halogen bonding is shown as blue dashed lines.

Analysis of full interaction maps (FIM) has been carried out to evaluate the probability of occurring hydrogen and halogen bonds around specific functional groups, and to reveal if the most likely combination of HB and XB donors and acceptors are satisfied in the crystal structure of compounds (**I**–**VI**). By applying the FIM tool implemented in the graphical user interface of the Mercury program (CSD version 2020.2.0, Materials module), this methodology generates, for the different functional groups of a molecule, 3D scatterplots through CSD contact searches with a chosen probe. These scatterplots are then converted into scaled density maps, which are combined for the whole molecule, resulting in different contour surfaces. Plotting contour surfaces shows the probability of an interaction at a certain grid-point with respect to random chance [64,65]. 3D visualization of how the probability of HB and XB interactions affects the overall packing in the crystal structure of compound (**VI**), which has been selected as a representative example, as depicted in Figure 7. FIMs for compounds (**I**–**V**) can be found in Figure S1 in the Supporting Information.

FIMs were calculated around the content of the asymmetric unit of each compound. The set of probe functional groups used as proxies of acceptors and donors of hydrogen bonds comprises oxygen atoms of carbonyl and water molecules (red), and uncharged and charged N–H (blue), respectively. C−Br (violet) was used as a probe of halogen bonds. Visual inspection of the interaction maps shows that the most expected positions of HB acceptors and donors and XB acceptors correlate well with the previously discussed packing arrangements of compounds (**I**–**VI**). In particular, all the FIMs show the strongest donor peak pointing at the O1 atom of the carbonyl group, which is the most expected hydrogen-bond acceptor. The presence of weak acceptor peaks (violet) along the C–Br bond axis for the 5-bromocytosinium cations indicates the moderate propensity of these ions to form halogen bonds.

### 2.3. Hirshfeld Surface Analysis

**Figure 8:**
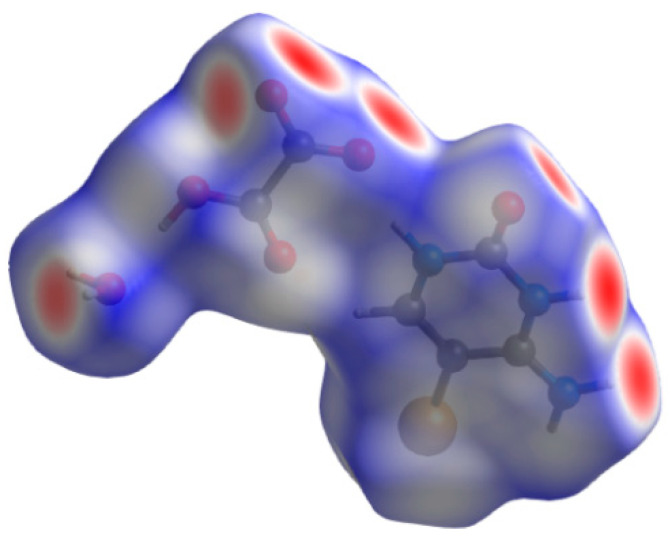
Visualization of the Hirshfeld surface for compound (**VI**) mapped on *d*_norm_. The color range is in arbitrary units; color scale: −0.7300 (red) 0.9540 a.u. (blue).

The intermolecular interactions were analyzed using the Hirshfeld surface (HS), which maps the normalized contact distance, *d*_norm_, on the pro-molecule surface, and its respective 2D fingerprint plots. From the HS, both the previously noted hydrogen and halogen bond may be estimated qualitatively from the intensity of the red spots corresponding to the distance between the interaction sites *d*_norm_ shorter than the sum of the vdW radii of interacting atoms. The intensity of the red spot varies from high, due to strong hydrogen bond, to feeble, due to the close contact by C=O^…^Br. The HS of compound (**VI**), which has been selected as a representative example, as depicted in Figure 8. HS for compounds (**I**–**V**) can be found in Figure S8 in the Supporting Information.

**Figure 9:**
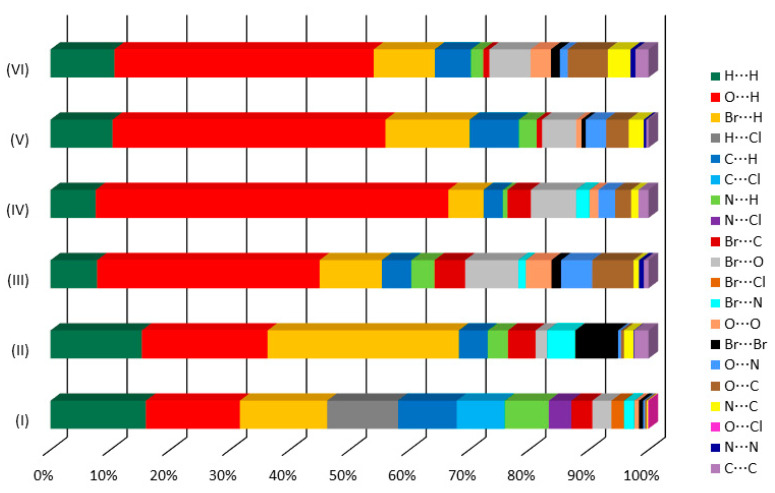
The relative contributions of different intermolecular contacts to the Hirshfeld surface area observed in the crystal packing of compounds (**I**–**VI**).

Figure 9 represents the relative contribution of the various atom···atom contacts observed for all the investigated crystal structures from the analysis of decomposed 2D-fingerprint plots (see Figures S2–S7 in the Supporting Information). In all cases, supramolecular arrangements are determined primarily by interatomic contacts involving oxygen and hydrogen atoms, which vary from 15.7% in compound (**I**) to 59.0% in compound (**IV**) and confirms that intermolecular neutral and charged hydrogen bonds play a fundamental role in the crystal packing. HS of compounds (**III**–**VI**) shows the maximum amount of O^…^H/H^…^O interactions compared to structures (I and II). These interactions account for 37.1–59.0% of the surface, in accordance with the presence of oxygenated anions. The H · · · H contacts, representing van der Waals interactions, comprise 7.6–16.0% of the total intermolecular contacts. The lower proportion of the Br · · · H/H · · · Br, C · · · H/H · · · C and Br · · · O/O · · · Br makes up 5.9–32.0, 3.2–9.8 and 2.0–8.9%, respectively, of the Hirshfeld surface. Lower abundances are found for N · · · H/H · · · N (0.8–7.4%), Br · · · Br (0.7–7.1%), O · · · C/C · · · O (0.5–6.9%), O · · · N/N · · · O (0.3–5.2%), Br · · · C/C · · · Br (0.9–4.6%) and Br · · · N/N · · · Br (1.2–4.6%). Cl · · · H/H · · · Cl (11.9%), C · · · Cl/Cl · · · C (8.0%), N · · · Cl/Cl · · · N (3.8%) and Br · · · Cl/Cl · · · Br (2.1%) are observed only for compound (**I**). Other intermolecular contacts contribute less than 4% to the Hirshfeld surface mapping.

### 2.4. Crystallographic Evidence of Proton Transfer in Compounds (***I***–***VI***)

**Figure 10:**
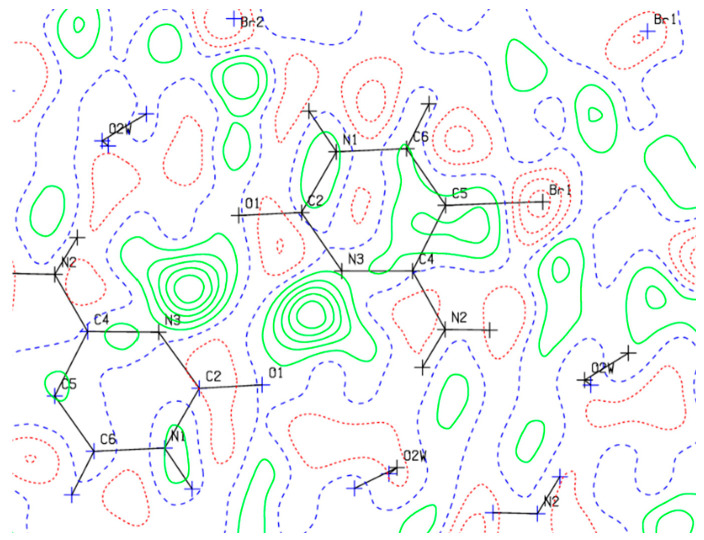
Visualization of the difference Fourier map through C2, N3, and C4 atoms of compound (**II**), revealing the hydrogen atom at the N3 ring position. Electron density is in the range −0.20 (blue line)–0.60 e/Å^3^ (green line).

Careful inspection of difference Fourier maps, calculated without the H3 hydrogen atom, gives clear evidence of protonation of compounds (**I**–**VI**) at the N3 ring position. Figure 10 shows the difference Fourier map for compound (**II**), which has been selected as a representative example, highlighting the electron density maximum corresponding to the position of the hydrogen atom in the neighborhood of N3 atom for pyrimidinium ring. Difference Fourier maps for compounds (**I**) and (**III**–**VI**) can be found in Figure S9 in the Supporting Information.

The effect of N3-protonation on the ring molecular geometries is revealed by inspection of Table 7. Comparison of selected geometrical parameters of compounds containing the planar 5-bromocytosinium cation shows that the independent molecules have almost equivalent geometry, as the differences among the corresponding bond distances and angles are in the range 0.019–0.040 (13) Å and 2.0–3.7 (10)°, respectively (hereafter the standard uncertainties of the differences have been calculated as the square root of the sum of the squares of the estimated standard deviations of geometrical parameters reported in Table 7, [66]). The N3 protonation reflects in the C4–N3–C2 bond angle, which is significantly larger than the corresponding one, 120.2° (6), for neutral 5-bromocytosine, BRCYTS [25], by 4.5–6.5 (8)°. This difference is fully consistent with the VSEPR theory, according to which the steric requirements of the lone pair on deprotonated aza nitrogen atom implies a wider region than the covalent bond N^+^−H. As a result, the protonation of the endocyclic N3 imino atom increases the C4–N3–C2 bond angle with respect to that on the unprotonated N3 atom [50].

**Figure 11:**
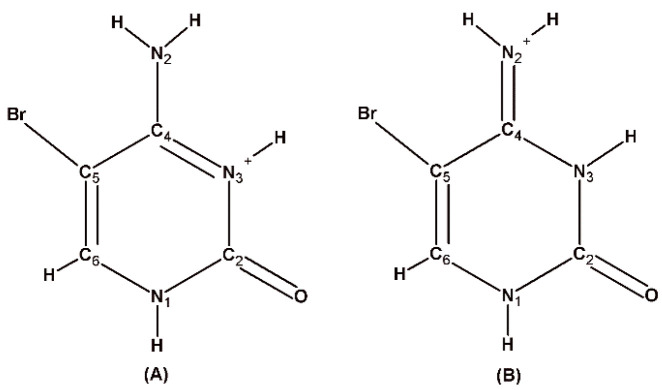
Two possible canonical forms (**A**,**B**) for 5-bromocytosinium cations in compounds (**I**–**VI**).

Due to protonation at the N3 atom, some degree of charge delocalization is expected, and can be revealed by checking the molecular geometry of the N3−C4(−N2)−C5 fragment of 5-bromocytosinium cations against that of the neutral 5-bromocytosine. In passing from BRCYTS to protonated compounds (**I**–**VI**), the most relevant distortions in the molecular geometry of the fragment can be summarized as follows: (i) A decrease of the N2-C4 bond distances by 0.037–0.058 (9) Å); (ii) an increase of the N3−C4 bond distances by 0.004–0.023 (6) Å*;* (iii) a decrease of the N3-C4-C5 bond angle by 2.0–4.3 (7)° (in BRCYTS the corresponding bond distances and angle are 1.351, 1.337 Å and 120.2°, respectively). Thus, the observed structural changes, although to the limits of statistical relevance, taken together suggest that protonation at the N3 ring position causes a redistribution of π-electron density in the cation, increasing π-donation from the amino group to the pyrimidinium ring (canonical form (B) in Figure 11). Planar arrangement of the fragment (the maximum deviation from the least-squares plane being 0.004 (1) Å for the N2 atom of the amino group) is another evidence of the proposed π-delocalization. Raising π-donation from the amino group to the pyrimidine ring favors, as observed in the above-reported discussion of crystal packing, the N_(amino)_–H^…^X hydrogen bonds in compounds (**I**–**VI**) to become progressively stronger, as the positive charge which resides on the nitrogen atom increases the polarity of the N–H bonds [67,68].

## 3. Materials and Methods

### 3.1. Supramolecular Synthesis

5-Bromocytosine, oxalic and phosphoric acids were purchased from Aldrich (99% purity) and subjected to further purification by successive sublimation under reduced pressure. Mineral acids were used as received without any further purification. For each compound, 0.1 mmol of 5-bromocytosine was dissolved in water. The solutions were warmed over a heating magnetic stirrer hotplate at 60 °C with continuous stirring for 12 h under reflux. For compounds (**I**–**IV**), water solutions of the diluted mineral acids were then added, while stirring, dropwise until pH reached 2. For compounds (**V**) and (**VI**), equimolar distilled water solutions of phosphoric and oxalic acids were then added while stirring. The resulting solutions were allowed to cool slowly to room temperature and then filtered. After ca one week, plate-shaped colorless crystals were obtained by slow evaporation of the solvent at room temperature.

### 3.2. Single Crystal Structure Analysis

The single crystal structures were examined on an Xcalibur S CCD diffractometer (graphite-monochromated Mo Kα radiation, λ = 0.710689 Å, Oxford Diffraction, Oxford, UK) at room temperature using the *CrysAlisPro* software package (Rigaku Oxford Diffraction, Yarnton, England, 2018) [69]. The crystal structures were solved by direct methods using SIR2004 [70]. All the nonhydrogen atoms were refined anisotropically by the full-matrix least-squares method based on squared structure factors (*F*^2^) using SHELXL-2014/7 [71], within the WinGX system [72]. All H atoms were found from difference Fourier, but for final refinement, all C-bound H atoms were placed in calculated positions, with C-H = 0.97 Å and *U*_iso_ (H) = 1.2 U_eq_ of the parent C atom and treated as riding on the adjacent atoms. Positional and isotropic thermal parameters of H atoms of the heteroatoms were freely refined in all but compounds (**I**), (**II**), (**IV**), and (**V**). For these compounds, free refinement of positional coordinates of atoms of the amino groups resulted in an unsatisfactory wide range of N−H distances, and consequently, these bond lengths were restrained to 0.86 Å. The molecular and packing diagrams were prepared using the Mercury 3.9 program package [73]. Difference Fourier maps were computed using Platon. CCDC 2073401-2073406 contains the supplementary crystallographic data for this paper. These data can be obtained free of charge via http://www.ccdc.cam.ac.uk/conts/retrieving.html (accessed on 26 March 2021) or from the CCDC, 12 Union Road, Cambridge CB2 1EZ, UK; Fax: +44 1223 336033; E-mail: deposit@ccdc.cam.ac.uk).

The details of crystal data, data collection, and structure refinement can be found in Table S16 of the Supporting Information.

### 3.3. Computational Methods

The Hirshfeld surfaces (HS) and their associated 2D fingerprint plots were computed using *Crystal Explorer17* (University of Western Australia: Crawley, AU, Australia [74]).

Quantum chemical calculations were performed on a series of selected clusters constructed from the asymmetric unit of each compound and extended to properly account for the hydrogen and halogen bonds located based on the X-ray structural analysis.

All the calculations were carried out—adopting the following protocol for each cluster. First, the structure, once extracted from the crystal structure, was partially optimized by keeping frozen the relative roto-translations between the different moieties (obviously in the case of monoatomic ions, only the relative distance was constrained). In this case, only the bond distances and the intramolecular bond angles were allowed to relax. This procedure was carried out using Hartree–Fock calculations with the 6-31+G(d) basis set. It is important to remark that further test-optimizations in the framework of Density Functional Theory, using the wB97XD functional, including the dispersion correction [75] with the same basis set, carried out on small clusters, did not significantly improve the final outcome, essentially because of the intramoieties constraints, and hence, not utilized on larger clusters. Subsequently, QTAIMS analysis was conducted on single points (SP) calculations at the wB97XD /6-31+G(d) level of theory on the optimized structures. The software Gaussian 09 [76] was utilized for the optimization and SP calculations, whereas the software Multiwfn, version 3.7 [77] was adopted for the QTAIMS analysis. All the optimized structures and QTAIMS additional details not reported in this study can be found in the Supplementary Information. Cartesian coordinates, coordinates and index of the bond CP for all the cluster adopted in this work can be found in Table S15 of the Supporting Information.

## 4. Conclusions

In summary, six novel organic salts of 5-bromocytosine were prepared and fully characterized by single crystal X-ray diffraction. In addition to X-ray analyses, the topological analysis based on the QM and subsequent QTAIMS calculations have confirmed that all the experimentally identified short-range interactions correspond to bond CP (i.e., critical points with rank and signature equal to 3 and −1, respectively) at which interaction energies are sensibly larger, and in some cases much larger, than the thermal energies.

At variance with pure 5-bromocytosine, in which halogen bonding interactions are not present, and the molecular assembly is dominated by conventional hydrogen bonds, all but (**II**) compounds show weak to moderately strong C–Br^…^O interactions (Br^…^O distances are from 4% to 24% shorter than the sum of *R*_vdW_ and the calculated interaction energies are in the range of ≈ 1.0–4.0 kcal mol^−1^).

Although halogen bonding plays an appreciable role, and in the absence of base stacking interactions between consecutive 5-bromocytosinium ions, in all six compounds, the crystal packing is mainly stabilized by conventional hydrogen bonds. Most of these HB interactions are due to strong hydrogen-bond donor N−H, N^+^−H and O−H sites in the 5-bromocytosinium cation, in the counterions, and in the water and H_3_O^+^ molecules.

In compounds (**I**), (**III**), and (**IV**), C–Br^…^O halogen bonds involve the carbonyl oxygen atoms of adjacent 5-bromocytosinium ions as XB acceptors, while in (**V**) and (**VI**), oxygen atoms of the counterions (dihydrogen monophosphate and hydrogen oxalate, respectively) act as XB acceptors.

**Figure 12:**
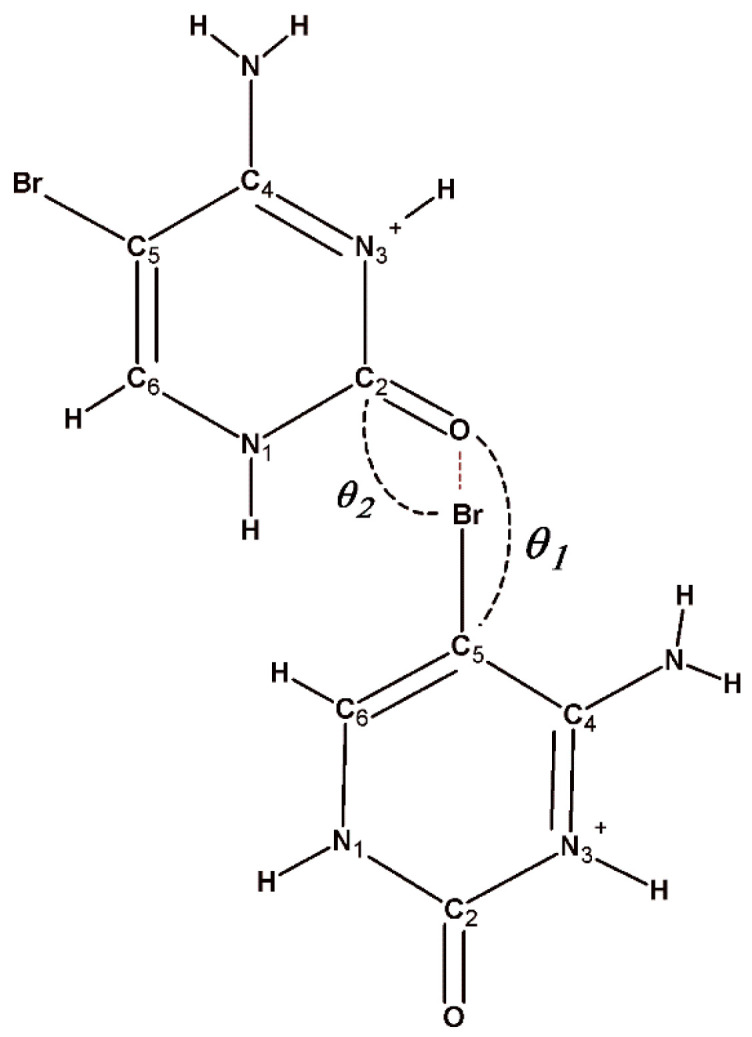
Schematic showing angles defining the approach of the *lp* acceptor towards the *lp* donor (*θ*_1_) and the *lp* donor towards the *lp* acceptor (*θ*_2_).

In compound (**III**), showing two weak “lateral” halogen interactions (*R*_XB_ = 0.94 and 0.90, respectively), the C–Br^…^O angles, *θ*_1_, are 150.3 (2)° and 157.0 (2)°, and the C=O^…^Br angles, *θ*_2_, are 150.9 (2)° and 157.6 (2)° (Figure 12). Similar values for the corresponding *θ*_1_ and *θ*_2_ angles have been found in 5-chlorouracil/melamine (2:1), 5-bromouracil/melamine (2:1) and 5-bromo-1-methyl uracil/melamine (2:1) complexes [54]. These values indicate significant deviations from the average value observed for *θ*_2_, 113°, in 5-halonucleobases [9]. They could suggest that strong linear XB with carbonyl oxygen atoms favors a halogen···O=C angle close to 120°, corresponding to the optimum approach of the halogen atoms to the sp^2^ orbitals on carbonyl oxygen atoms. Conversely, in the presence of weak XB, *θ*_1_ deviates from linearity, and *θ*_2_ becomes more sensitive to additional interactions competing for the O acceptor site.

Such experimental/theoretical study may provide useful information on the stability of the DNA model containing protonated halonucleobases—in which the pyrimidinium N^+^–H moiety would act as an electron-withdrawing group to activate the C–Br moiety as XB donor. Indeed, although electrostatic characteristics of halonucleobases are different from those of DNA (due to the presence of the phosphate backbone), our results suggest that halogenated nucleic acids could use the carbonyl oxygen atom of 5-bromocytosine, besides oxygen atoms of the phosphate group [3,6,13,14], as favorable negative site able to interact with the positive σ-hole on halogen atoms.

## Figures and Tables

**Table 1:** Hydrogen-bond geometry (Å, °) and topological analysis for compound (**I**). E_int_ is reported in kcal/mol. For additional details concerning QTAIMS results see Table S1 in the Supporting Information.

*D*—H···*A*	*D*—H	H···*A*	*D*···*A*	*D*—H···*A*	−G(r)/V(r)	E_int_
N2—H2A···Cl1	0.86	2.63	3.400 (7)	150.1	*1.07*	*3.0*
N2—H2B···Cl1C	0.86	2.40	3.224 (6)	160.9	*1.01*	*5.1*
N1—H1···O1B	0.81 (10)	2.01 (10)	2.808 (8)	169 (11)	*0.98*	*9.2*
N3—H3···Cl1	0.86	2.41	3.230 (6)	159.5	*0.94*	*7.2*
N2A—H2A1···Cl1A	0.86	2.39	3.163 (7)	149.1	*0.98*	*5.2*
N2A—H2A2···O1A ^i^	0.86	2.58	3.379 (9)	155.9	*1.16*	*1.6*
N1A—H1A···Cl1 ^ii^	0.86	2.63	3.375 (7)	146.1	*1.08*	*3.5*
N3A—H3A···Cl1A	0.83 (8)	2.28 (8)	3.062 (6)	160 (8)	*0.88*	*8.1*
N2B—H2B1···O2_w_	0.86	2.06	2.862 (9)	154.1	*0.94*	*7.2*
N2B—H2B2···Cl1A	0.86	2.39	3.201 (6)	157.6	*0.99*	*5.7*
N1B—H1B···O1	0.86	1.98	2.822 (8)	164.3	*0.98*	*9.1*
N3B—H3B···Cl1B	0.84 (9)	2.24 (9)	3.078 (6)	171 (9)	*0.81*	*10.1*
N2C—H2C1···Cl1C	0.86	2.34	3.119 (7)	151.7	*0.94*	*6.6*
N1C—H1C···Cl1B ^iii^	0.86	2.40	3.113 (7)	140.1	*1.08*	*2.6*
N3C—H3C···Cl1C	0.86	2.31	3.110 (6)	154.8	*0.90*	*7.3*
O2_w_—H2A···Cl1B	0.89 (12)	2.28 (12)	3.056 (7)	146 (10)	*0.95*	*6.5*
O2_w_—H2B···Cl1 ^iv^	0.71 (8)	2.46 (8)	3.156 (7)	171 (9)	*0.95*	5.1

Symmetry codes: ^i^ *x*, −*y*−1/2, *z* + 1/2; ^ii^ −*x*, *y* − 1/2, ^iii^ −*x*, *y* − 1/2; ^iv^ −*z* + 5/2, *x*, *y*, *z* + 1.

**Table 2:** Hydrogen-bond geometry (Å, °) and topological analysis for compound (II). E_int_ is reported in kcal/mol. For additional details concerning QTAIMS results see Tables S2–S4 in the Supporting Information.

D—H···A	D—H	H···A	D···A	D—H···A	−G(r)/V(r)	E_int_
N2—H2A···O2_w_^i^	0.86	2.00	2.831 (4)	163.4	0.96	6.2
N2—H2B···O2_w_	0.86	2.09	2.881 (4)	152.3	0.99	7.5
N1—H1···Br2	0.83 (4)	2.43 (4)	3.268 (3)	179 (3)	0.88	5.3
N3—H3···O1 ^ii^	0.86 (4)	1.91 (4)	2.754 (4)	171 (4)	1.04	8.6
O2_W_—H2A···Br2 ^iii^	0.73 (5)	2.51 (5)	3.241 (4)	173 (5)	0.97	4.6
O2_W_—H2B···Br2 ^iv^	0.93 (6)	2.61 (6)	3.475 (3)	155 (5)	1.08	3.0

Symmetry codes: ^i^ −*x* + 1, *y*, −*z* − 1/2; ^ii^ −*x*, *y*, −*z* − 1/2; ^iii^ −*x* + 1/2, −*y* + 1/2, −*z*; ^iv^ *x* + 1, *y*, *z*.

**Table 3:** Hydrogen-bond geometry (Å, °) and topological analysis for compound (**III**). E_int_ is reported in kcal/mol. For additional details concerning QTAIMS results see Tables S5–S7 in the Supporting Information.

*D*—H···*A*	*D*—H	H···*A*	*D*···*A*	*D*—H···*A*	−G(r)/V(r)	E_int_
N2—H2A···O3	0.83 (3)	2.03 (3)	2.851 (3)	173 (3)	0.96	7.7
N2—H2B···O5_w_^i^	1.00 (5)	1.89 (5)	2.887 (4)	175 (4)	1.00	6.3
N1—H1···O4C ^ii^	0.79 (3)	2.53 (3)	3.306 (4)	171 (3)	1.02	3.0
N3—H3···O2	0.92 (3)	1.91 (3)	2.824 (3)	176 (3)	0.95	8.5
C6—H6···O1C ^ii^	0.97	2.33	3.226 (4)	154.2	1.21	1.8
N2C—H2CA···O3	0.84 (4)	2.11 (4)	2.926 (4)	165 (3)	0.95	5.7
N2C—H2CB···O3C	0.84 (3)	2.16 (3)	2.981 (4)	166 (3)	0.93	4.6
N1C—H1C···O3C ^i^	0.73 (3)	2.38 (3)	2.984 (3)	142 (3)	1.07	2.6
N1C—H1C···O4C ^i^	0.73 (3)	2.33 (3)	3.028 (4)	163 (3)	1.01	3.0
N3C—H3C···O2C	0.97 (3)	1.78 (3)	2.747 (3)	175 (3)	0.95	8.5
C6C—H6C···O4 ^i^	0.97	2.47	3.385 (3)	157.9	1.2	1.8
O5_w_—H52···O1	0.89 (1)	2.01 (2)	2.869 (4)	164 (4)	1.01	6.7
N2A—H2AA···O3A	0.89 (3)	1.98 (3)	2.864 (3)	173 (3)	0.96	7.8
N2A—H2AB···O6_w_^iii^	0.85 (4)	2.08 (4)	2.918 (4)	169 (4)	1.07	2.4
N1A—H1A···O2B ^iv^	0.77 (3)	2.32 (3)	2.909 (3)	134 (3)	1.07	2.7
N1A—H1A···O4B ^iv^	0.77 (3)	2.60 (3)	3.355 (4)	167 (3)	1.02	2.9
N3A—H3A···O2A	0.76 (3)	2.08 (3)	2.835 (3)	175 (3)	0.95	6.1
C6A—H6A···O1B ^iv^	0.97	2.25	3.166 (3)	158.1	1.2	1.8
N2B—H2BA···O3A	0.88 (4)	2.07 (4)	2.921 (3)	164 (3)	0.93	4.6
N2B—H2BB···O3B	0.84 (3)	2.16 (3)	2.991 (4)	168 (3)	0.94	6.8
N1B—H1B···O4B ^iii^	0.80 (3)	2.28 (3)	3.040 (4)	159 (3)	1.07	2.7
N3B—H3B···O2B	0.85 (3)	1.93 (3)	2.764 (3)	168 (3)	0.92	7.7
C6B—H6B···O4A ^iii^	0.97	2.39	3.336 (3)	165.1	1.21	1.7
O6_w_—H61···O1A	0.90(1)	1.99 (1)	2.880 (3)	172 (4)	1.00	6.1
O6_w_—H62···O2A	0.89 (1)	2.01 (2)	2.838 (3)	153 (4)	0.98	6.8

Symmetry codes: ^i^ *x* − 1, *y*, *z*; ^ii^ *x*, *y*, *z* + 1; ^iii^ *x* + 1, *y*, *z*; ^iv^ *x*, *y*, *z* − 1.

**Table 4:** Hydrogen-bond geometry (Å, °) and topological analysis for compound (**IV**). E_int_ is reported in kcal/mol. For additional details concerning QTAIMS results see Tables S8–S10 in the Supporting Information.

*D*—H···*A*	*D*—H	H···*A*	*D*···*A*	*D*—H···*A*	−G(r)/V(r)	E_int_
N2—H2A···O2	0.86 (3)	2.08 (3)	2.883 (2)	155 (3)	0.91	4.8
N2—H2B···O4 ^i^	0.86 (3)	2.15 (3)	2.924 (2)	150 (2)	0.92	6.4
N1—H1···O4 ^ii^	0.82 (3)	2.11 (3)	2.877 (2)	154 (3)	0.93	5.0
N3—H3···O3	0.77 (3)	2.06 (3)	2.831 (2)	174 (3)	0.86	6.0
O6_w_—H6A···O2 ^i^	0.79 (3)	1.77 (3)	2.560 (2)	174 (3)	0.94	14.0
O6_w_—H6B···O3 ^iii^	0.89 (3)	1.70 (3)	2.588 (2)	173 (3)	0.79	17.3
O6_w_—H6C···O5 ^iv^	0.93 (3)	1.58 (4)	2.504 (2)	177 (3)	0.55	25.6

Symmetry codes: ^i^ −*x* + 1, −*y* + 1, −*z* + 2; ^ii^ −*x*, *y* − 1/2, −*z* + 5/2; ^iii^ −*x*, −*y* + 1, −*z* + 2; ^iv^ *x*, *y*, *z* − 1.

**Table 5:** Hydrogen-bond geometry (Å, °) and topological analysis for compound (**V**). E_int_ is reported in kcal/mol. For additional details concerning QTAIMS results see Tables S11 and S12 in the Supporting Information.

*D*—H···*A*	*D*—H	H···*A*	*D*···*A*	*D*—H···*A*	−G(r)/V(r)	E_int_
N2—H2A···O3	0.86	1.91	2.769 (3)	177.2	0.94	11.2
N2—H2B···O1 ^i^	0.86	2.30	2.818 (3)	118.9	1.16	3.4
N1—H1···O3 ^ii^	0.87 (3)	1.80 (3)	2.661 (3)	171 (3)	0.95	9.9
N3—H3···O2	0.92 (3)	1.81 (3)	2.720 (3)	175 (3)	0.94	9.5
O4—H4···O2 ^iii^	0.83 (4)	1.74 (4)	2.564 (2)	172 (4)	0.97	9.3
O5—H5···O4 ^iv^	0.87 (5)	1.87 (5)	2.722 (3)	170 (5)	0.97	10.2

Symmetry codes: ^i^ *x* + 1, −*y* + 1/2, *z* − 1/2; ^ii^ *x* − 1, −*y* + 1/2, *z* + 1/2; ^iii^ *x* + 1, *y*, *z*; ^iv^ −*x* + 1, −*y* + 1, −*z*.

**Table 6:** Hydrogen-bond geometry (Å, °) and topological analysis for compound (**VI**). E_int_ is reported in kcal/mol. For additional details concerning QTAIMS results see Tables S13 and S14 in the Supporting Information.

*D*—H···*A*	*D*—H	H···*A*	*D*···*A*	*D*—H···*A*	−G(r)/V(r)	E_int_
N2—H21···O2 ^i^	0.89 (4)	1.89 (4)	2.770 (3)	171 (4)	0.91	7.3
N2—H22···O6_w_^ii^	0.85 (4)	2.57 (4)	3.389 (4)	162 (3)	1.18	2.0
N1—H1···O2	0.83 (4)	1.90 (4)	2.710 (3)	167 (4)	0.92	7.3
N3—H3···O3 ^i^	0.82 (4)	1.86 (4)	2.673 (3)	173 (4)	0.91	8.3
O5—H5···O6_w_	0.83 (5)	1.86 (5)	2.674 (4)	169 (5)	1.00	9.0
O6_w_—H61···O1 ^iii^	0.84 (7)	2.10 (7)	2.852 (4)	149 (6)	1.03	6.0
O6_w_—H62···O3 ^iv^	0.73 (5)	2.18 (5)	2.849 (4)	155 (5)	0.95	7.0

Symmetry codes: ^i^ *x* + 1, −*y* + 1/2, *z* − 1/2; ^ii^ -*x* + 1, −*y*, −*z* + 1; ^iii^ −*x*, *y* − 1/2, −*z* + 3/2; ^iv^ −*x*, −*y*, −*z* + 2.

**Table 7:** Selected geometric parameters of 5-bromocytosinium cation for compounds (**I**–**VI**), HABKEA ^a^ and NUZKEY ^b^.

*Bond distances* (Å)									
		**(I)**			**(II)**		**(III)**		
		**Mol A**	**Mol B**	**Mol C**			**Mol A**	**Mol B**	**Mol C**
*Br1—C5*	1.855 (7)	1.870 (8)	1.855 (7)	1.880 (7)	1.884 (3)	1.871 (3)	1.874 (2)	1.870 (3)	1.867 (3)
*O1—C2*	1.215 (8)	1.202 (9)	1.211 (8)	1.204 (9)	1.219 (4)	1.215 (3)	1.213 (3)	1.211 (3)	1.202 (3)
*N2—C4*	1.314 (9)	1.310 (9)	1.304 (9)	1.307 (10)	1.293 (4)	1.306 (4)	1.305 (3)	1.298 (4)	1.300 (4)
*N1—C6*	1.336 (9)	1.348 (10)	1.351 (9)	1.353 (9)	1.360 (5)	1.363 (4)	1.355 (3)	1.360 (4)	1.351 (4)
*N1—C2*	1.362 (9)	1.378 (10)	1.358 (9)	1.382 (9)	1.362 (4)	1.368 (4)	1.372 (4)	1.363 (3)	1.368 (4)
*C2—N3*	1.381 (9)	1.388 (10)	1.381 (9)	1.385 (10)	1.372 (4)	1.376 (4)	1.369 (4)	1.375 (3)	1.380 (4)
*N3—C4*	1.356 (8)	1.353 (10)	1.348 (9)	1.345 (9)	1.360 (4)	1.348 (3)	1.359 (3)	1.341 (3)	1.356 (4)
*C4—C5*	1.420 (9)	1.419 (10)	1.441 (9)	1.422 (10)	1.426 (4)	1.424 (4)	1.417 (3)	1.432 (3)	1.420 (4)
*C5—C6*	1.354 (10)	1.323 (10)	1.350 (10)	1.324 (10)	1.333 (5)	1.335 (4)	1.347 (4)	1.343 (4)	1.342 (4)
	**(IV)**	**(V)**	**(VI)**	**HABKEA**		**NUZKEY**			
					**Mol A**	**Mol B**	**Mol C**		
*Br1—C5*	1.874 (2)	1.876 (2)	1.871 (3)	1.871 (4)	1.867 (5)	1.859 (4)	1.887 (7)		
*O1—C2*	1.211 (2)	1.214 (3)	1.217 (3)	1.205 (5)	1.201 (8)	1.219 (8)	1.206 (9)		
*N2—C4*	1.304 (2)	1.305 (3)	1.309 (4)	1.309 (5)	1.305 (7)	1.298 (8)	1.293 (9)		
*N1—C6*	1.357 (3)	1.348 (3)	1.354 (3)	1.354 (6)	1.331 (8)	1.357 (8)	1.371 (11)		
*N1—C2*	1.368 (3)	1.371 (3)	1.369 (4)	1.368 (7)	1.364 (8)	1.348 (8)	1.361 (9)		
*C2—N3*	1.384 (2)	1.382 (3)	1.385 (4)	1.378 (5)	1.375 (7)	1.384 (8)	1.385 (11)		
*N3—C4*	1.352 (2)	1.348 (3)	1.355 (3)	1.353 (5)	1.355 (6)	1.348 (8)	1.341 (7)		
*C4—C5*	1.430 (2)	1.424 (3)	1.430 (4)	1.419 (7)	1.423 (7)	1.430 (8)	1.416 (9)		
*C5—C6*	1.351 (3)	1.350 (3)	1.353 (4)	1.349 (5)	1.352 (8)	1.351 (8)	1.331 (11)		
*Bond angles* (°)									
		**(I)**			**(II)**		**(III)**		
		**Mol A**	**Mol B**	**Mol C**			**Mol A**	**Mol B**	**Mol C**
*C6—N1—C2*	123.5 (6)	123.1 (6)	123.9 (6)	123.5 (6)	122.6 (3)	122.9 (3)	123.0 (3)	123.0 (3)	123.8 (3)
*O1—C2—N1*	123.8 (7)	125.0 (7)	124.0 (7)	123.8 (7)	122.7 (3)	123.3 (3)	122.6 (3)	123.5 (3)	124.0 (3)
*O1—C2—N3*	122.2 (6)	122.5 (7)	122.1 (6)	123.4 (7)	122.4 (3)	122.1 (3)	123.2 (3)	122.1 (2)	122.3 (3)
*N1—C2—N3*	114.0 (6)	112.5 (7)	114.0 (6)	112.8 (6)	115.0 (3)	114.6 (3)	114.2 (2)	114.4 (3)	113.7 (3)
*C4—N3—C2*	124.7 (6)	126.7 (6)	125.8 (6)	126.5 (6)	125.3 (3)	125.4 (3)	126.4 (2)	126.4 (2)	125.8 (2)
*N2—C4—N3*	117.9 (6)	118.9 (7)	119.3 (6)	119.1 (7)	119.5 (3)	119.2 (3)	118.9 (2)	119.6 (2)	118.4 (3)
*N2—C4—C5*	123.9 (6)	124.4 (7)	123.5 (6)	124.7 (7)	124.1 (3)	123.9 (3)	125.2 (2)	124.2 (3)	124.9 (3)
*N3—C4—C5*	118.2 (6)	116.6 (6)	117.2 (6)	116.3 (7)	116.3 (3)	116.9 (3)	115.9 (2)	116.2 (2)	116.8 (3)
*C6—C5—C4*	116.6 (7)	118.1 (7)	117.3 (7)	119.3 (7)	118.9 (3)	119.1 (3)	119.4 (2)	119.0 (3)	118.7 (3)
*C6—C5—Br1*	120.9 (5)	122.3 (6)	121.7 (5)	121.3 (6)	122.2 (2)	120.9 (2)	120.6 (2)	120.5 (2)	121.4 (2)
*C4—C5—Br1*	122.5 (5)	119.6 (5)	121.0 (5)	119.1 (6)	118.8 (2)	120.0 (2)	120.0 (2)	120.5 (2)	119.9 (2)
*N1—C6—C5*	122.6 (7)	123.0 (7)	121.8 (6)	121.4 (7)	121.6 (3)	121.0 (3)	121.0 (3)	121.0 (3)	121.2 (3)
	**(IV)**	**(V)**	**(VI)**	**HABKEA**		**NUZKEY**			
					**Mol A**	**Mol B**	**Mol C**		
*C6—N1—C2*	123.5 (2)	122.8 (2)	122.9 (2)	123.1 (4)	124.7 (5)	123.1(5)	122.5 (6)		
*O1—C2—N1*	124.1 (2)	123.4 (2)	122.9 (3)	123.6 (4)	125.9 (6)	125.5 (6)	123.5 (7)		
*O1—C2—N3*	121.9 (2)	121.6 (2)	122.0 (3)	122.5 (4)	120.9 (5)	119.7 (6)	121.9 (6)		
*N1—C2—N3*	114.0 (2)	114.9 (2)	115.1 (2)	113.9 (3)	113.1 (5)	114.8 (5)	114.6 (6)		
*C4—N3—C2*	125.8 (2)	124.8 (2)	124.7 (2)	126.3 (4)	126.0 (5)	125.7 (5)	125.8 (6)		
*N2—C4—N3*	119.2 (2)	119.0 (2)	119.4 (2)	118.8 (4)	119.1 (5)	119.4 (5)	120.0 (6)		
*N2—C4—C5*	123.7 (2)	123.5 (2)	123.2 (3)	124.7 (4)	124.2 (5)	124.3 (5)	123.6 (6)		
*N3—C4—C5*	117.1 (2)	117.4 (2)	117.3 (2)	116.5 (4)	116.7 (5)	116.3 (5)	116.4 (6)		
*C6—C5—C4*	118.1 (2)	118.4 (2)	118.4 (2)	118.6 (4)	118.0 (5)	118.5 (5)	120.0 (6)		
*C6—C5—Br1*	121.8 (1)	121.8 (2)	122.4 (2)	120.9 (3)	122.5 (4)	122.1 (4)	120.1 (5)		
*C4—C5—Br1*	120.0 (1)	119.8 (2)	119.2 (2)	120.6 (3)	119.5 (4)	119.4 (4)	119.9 (5)		
*N1—C6—C5*	121.5 (2)	121.4 (2)	121.4 (2)	121.5 (4)	121.1 (5)	121.2 (5)	120.7 (6)		

^a^ Ref. [26]. ^b^ Ref. [27].

## Data Availability

The data presented in this study are openly available in the Supplementary Information file.

## Supplementary Materials

The following are available online. Figure S1: full interaction maps for compounds (**I**–**V**); Figures S2–S7: Decomposed two-dimensional fingerprint plots; Figure S8: Hirshfeld surfaces for compounds (**I**–**V**); Figure S9: Difference Fourier maps for compounds (**I**) and (**III**–**VI**); Tables S1–S14: geometrical details, and related bond-CP index for the short-contact pair-interactions emerged from structural analysis; Table S15: Cartesian coordinates of all the clusters utilized in the work and Cartesian coordinates of the corresponding bond-CP. Table S16: Crystal data, data collection, and refinement for compounds (**I**–**VI**).

## Sample Availability

Samples of all compounds are available from the authors.
