# Supplementary material for: A Combined Experimental and Computational Study of Halogen and Hydrogen Bonding in Molecular Salts of 5-Bromocytosine
Source: Molecules. 2021 May 23;26(11):3111. doi: 10.3390/molecules26113111 (PMC8196974; doi:10.3390/molecules26113111)
Supplement: Supplementary file 1 [file molecules-26-03111-s001.zip › molecules-1190685-supplementary.pdf]

molecules

MDPI

Supporting Information

A Combined Experimental and Computational Study of Halogen and Hydrogen Bonding in Molecular Salts of 5-Bromocytosine

Massimiliano Aschi [1], Giorgia Toto Brocchi [2] and Gustavo Portalone [2,*]

[1] Dipartimento di Scienze Fisiche e Chimiche Università degli Studi di L'Aquila, Via Vetoio, 67100 Coppito, AQ, Italy
[2] Dipartimento di Chimica, Sapienza Università di Roma, P.le A. Moro 5, 00185 Roma, Italy
* Correspondence: gustavo.portalone@uniroma1.it (G.P.)

INDEX:

Figures S1–S6: Decomposed two-dimensional fingerprint plots for compounds (I)–(VI).

Tables S1–S14: Geometrical details, and related bond-CP index for the short-contact pair-interactions emerged from structural analysis.

Table S15: Cartesian coordinates of all the clusters utilized in the work and Cartesian coordinates of the corresponding bond-CP.

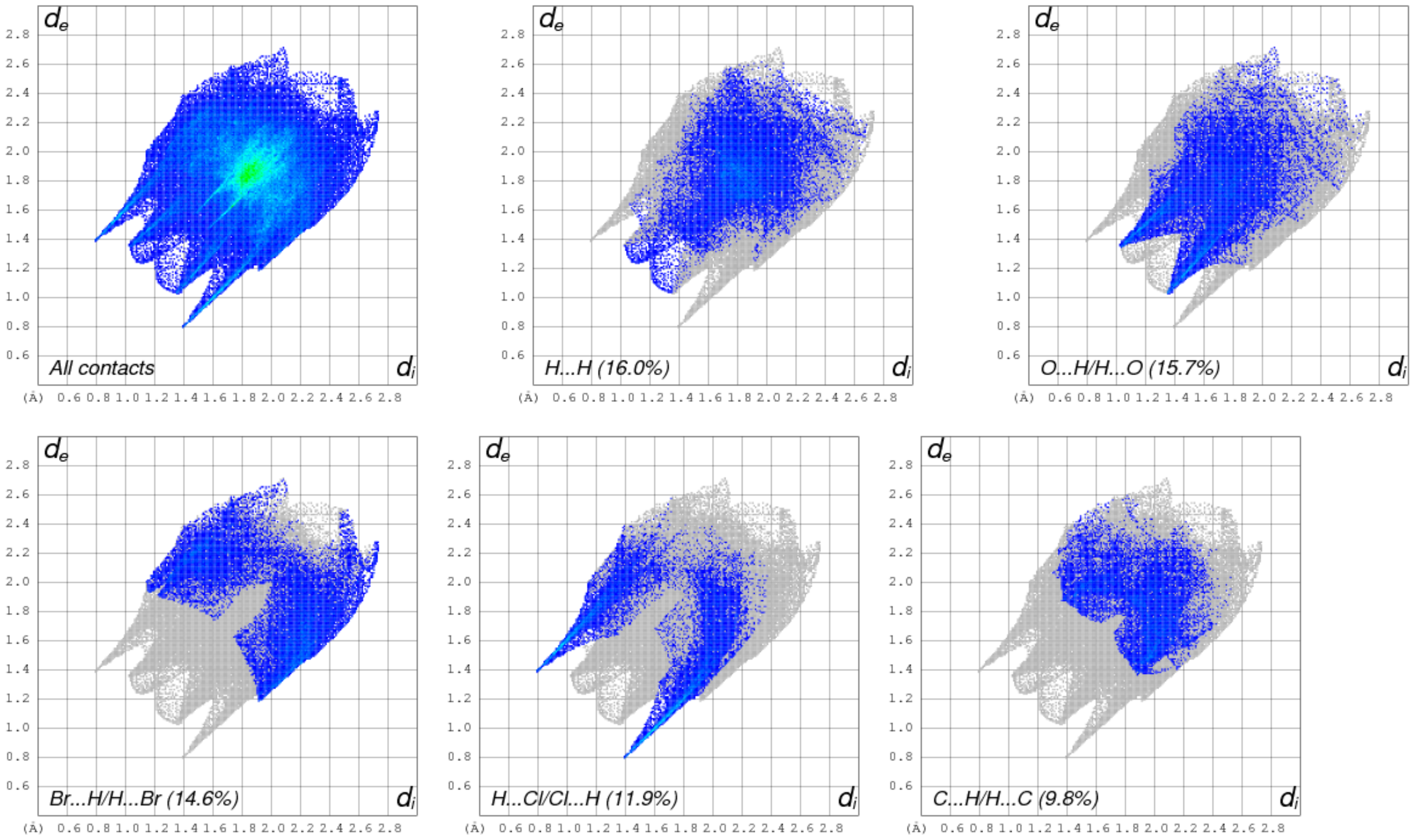

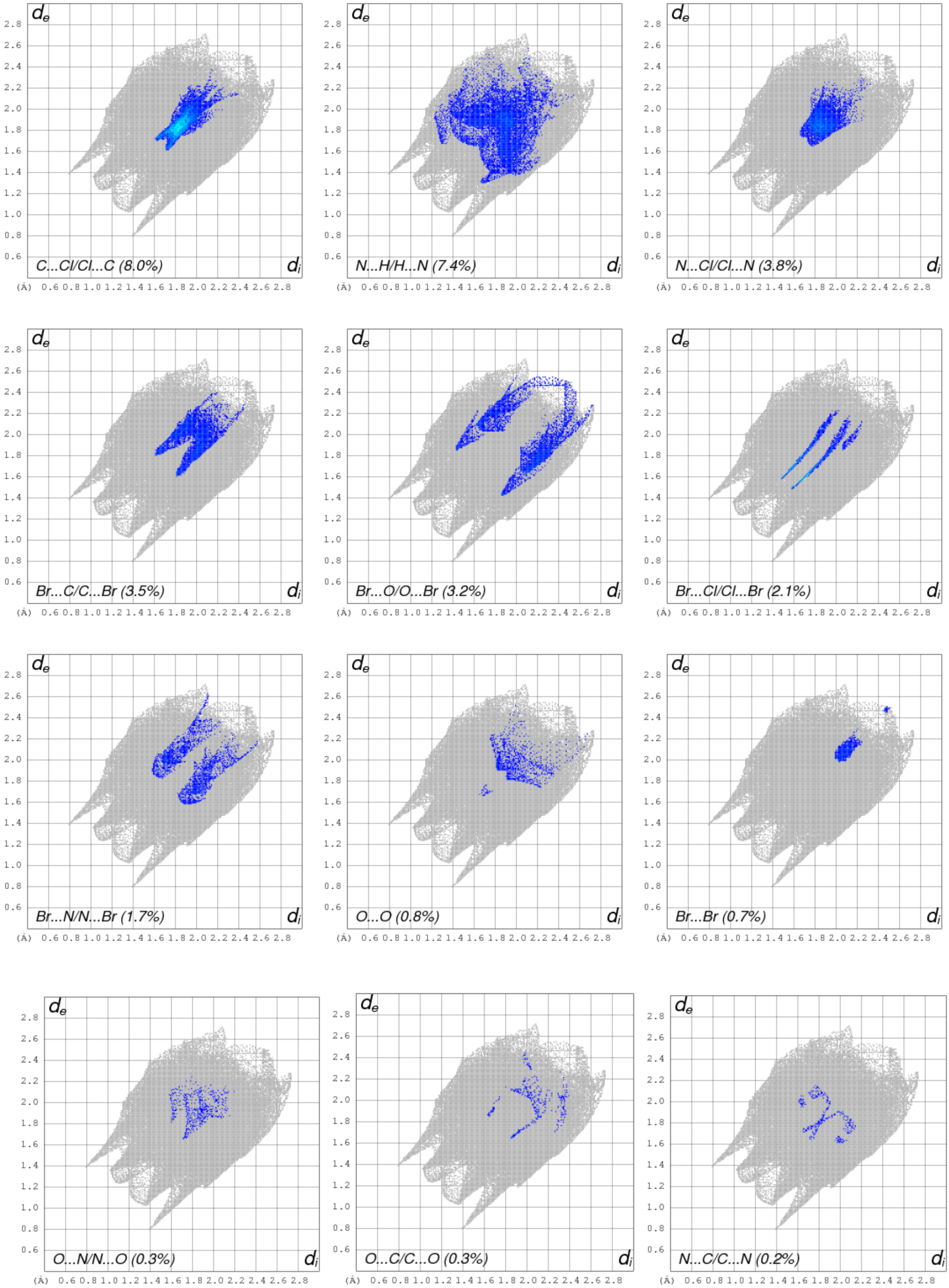
C...Cl/Cl...C (8.0%)
N...H/H...N (7.4%)
N...Cl/Cl...N (3.8%)
Br...C/C...Br (3.5%)
Br...O/O...Br (3.2%)
Br...Cl/Cl...Br (2.1%)
Br...N/N...Br (1.7%)
O...O (0.8%)
Br...Br (0.7%)
O...N/N...O (0.3%)
O...C/C...O (0.3%)
N...C/C...N (0.2%)

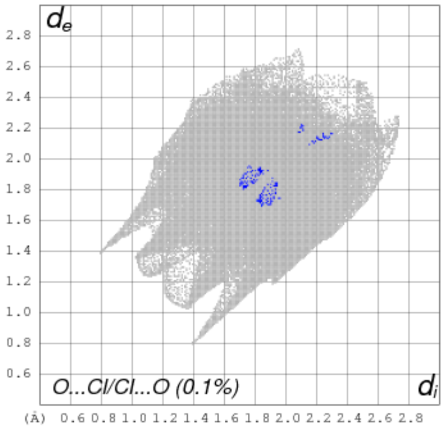

Figures S1: decomposed two-dimensional fingerprint plots for compound (I).

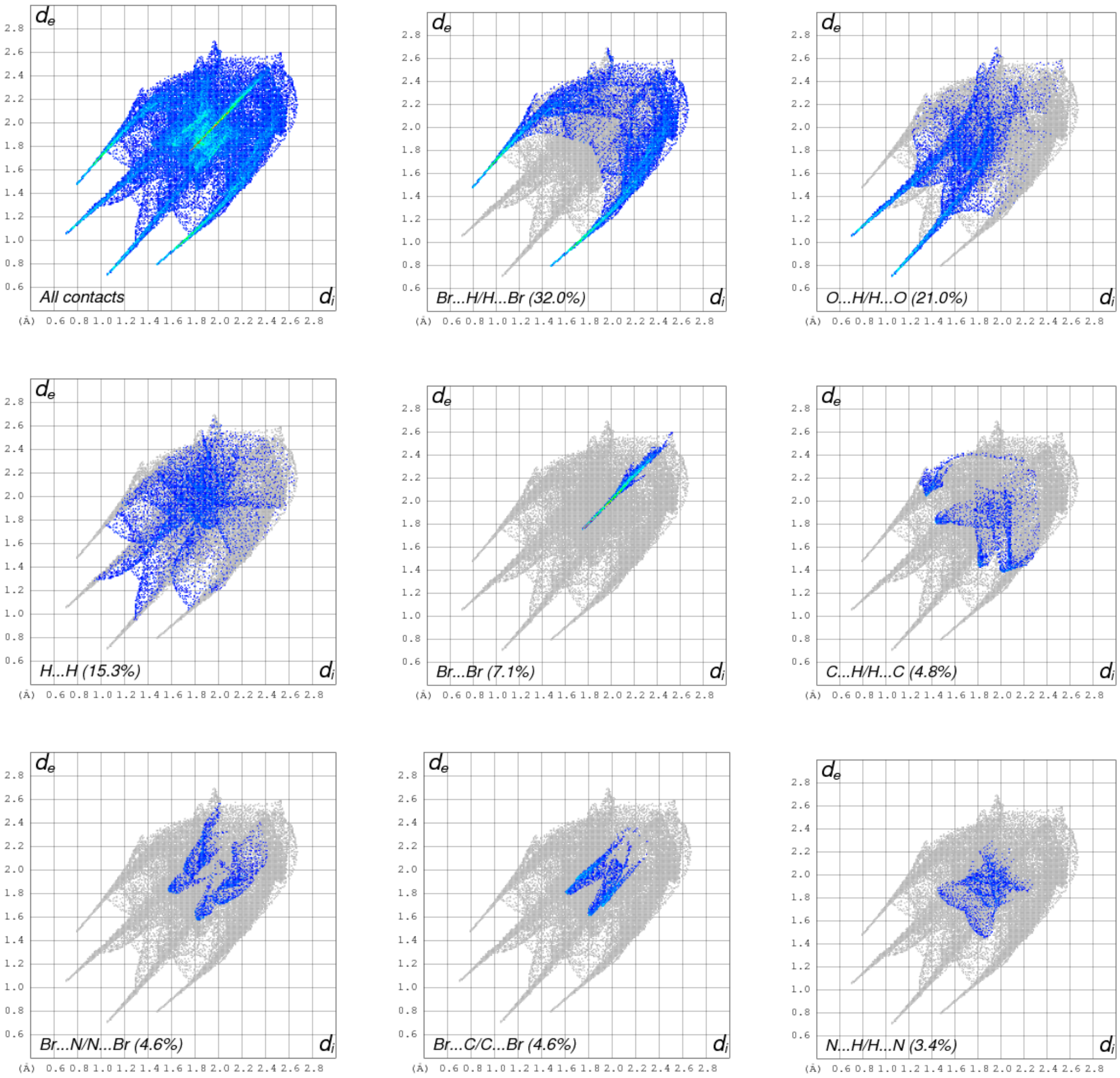
All contacts
Br...H/H...Br (32.0%)
O...H/H...O (21.0%)
H...H (15.3%)
Br...Br (7.1%)
C...H/H...C (4.8%)
Br...N/N...Br (4.6%)
Br...C/C...Br (4.6%)
N...H/H...N (3.4%)

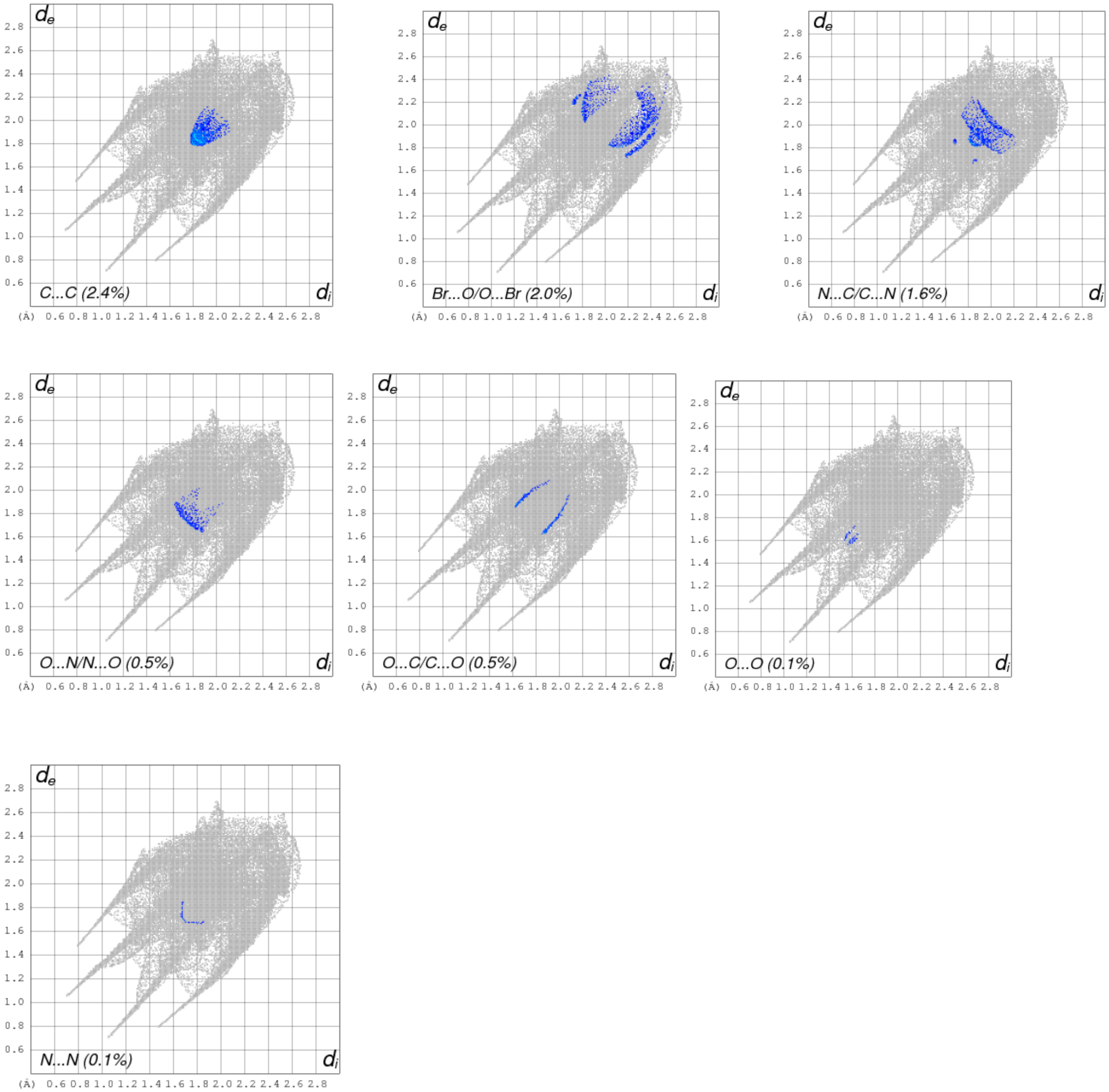

Figures S2: decomposed two-dimensional fingerprint plots for compound (II).

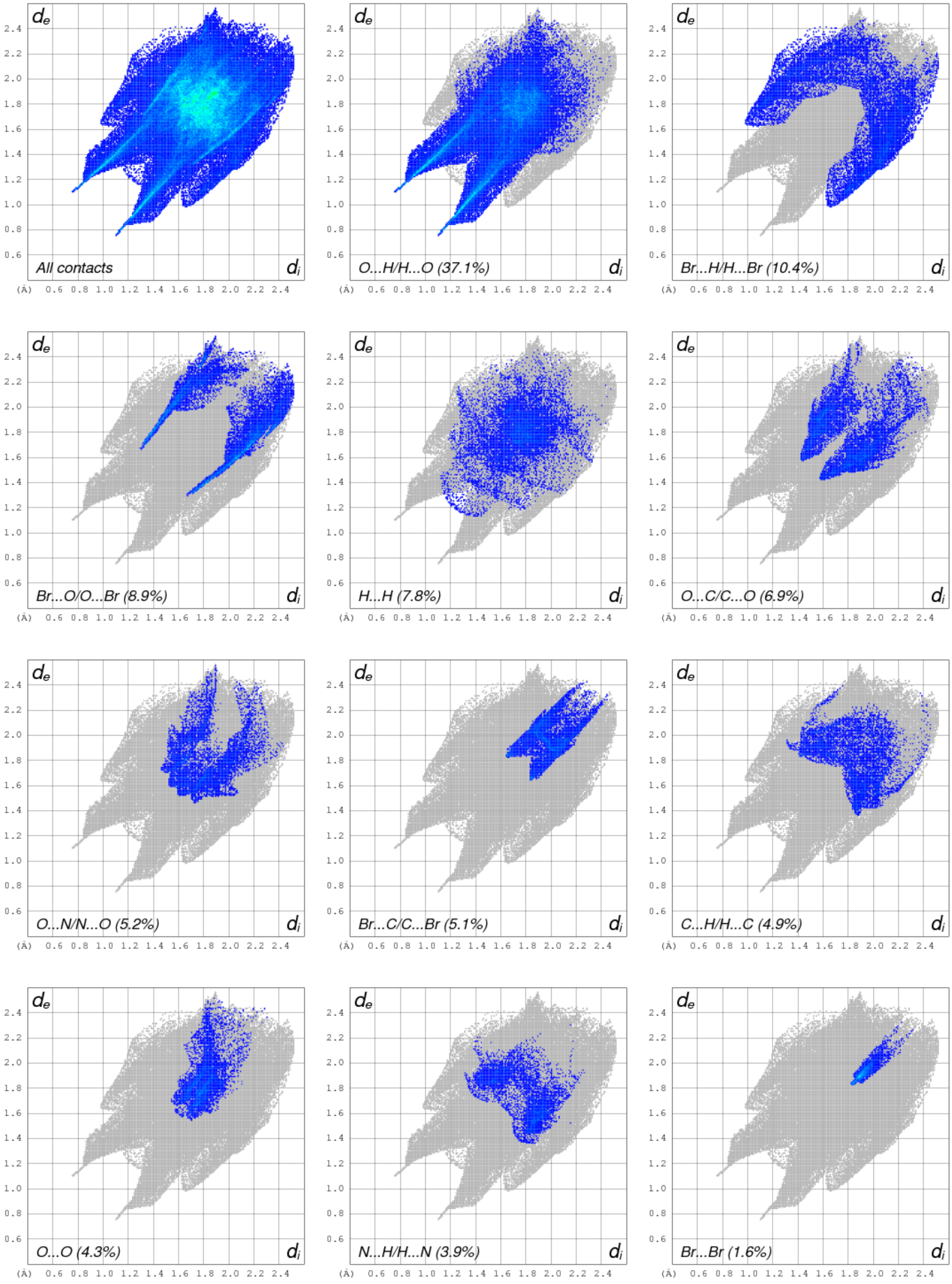
de
di
(Å)
0.6 0.8 1.0 1.2 1.4 1.6 1.8 2.0 2.2 2.4
All contacts
O...H/H...O (37.1%)
Br...H/H...Br (10.4%)
Br...O/O...Br (8.9%)
H...H (7.8%)
O...C/C...O (6.9%)
O...N/N...O (5.2%)
Br...C/C...Br (5.1%)
C...H/H...C (4.9%)
O...O (4.3%)
N...H/H...N (3.9%)
Br...Br (1.6%)

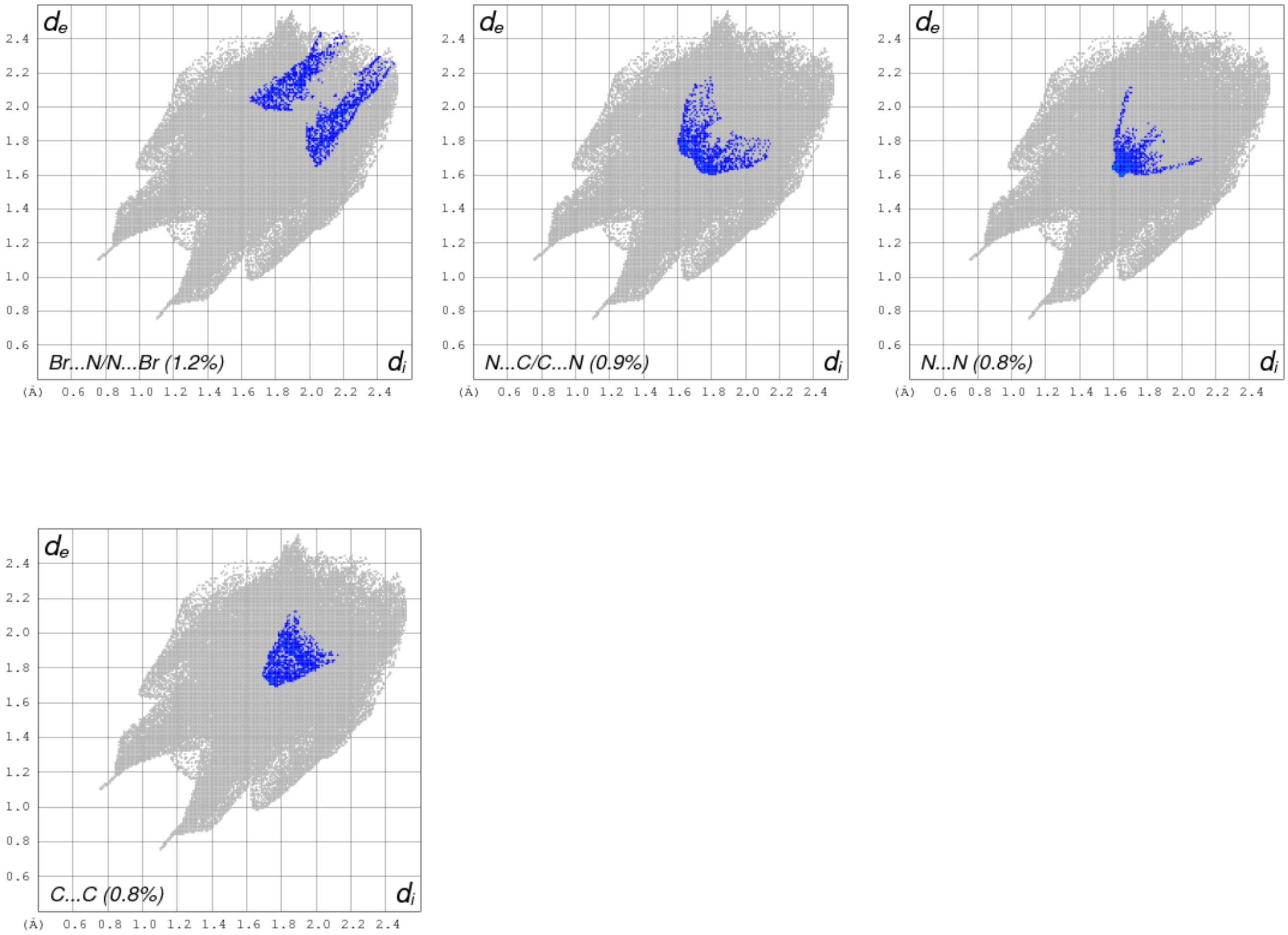

Figures S3: decomposed two-dimensional fingerprint plots for compound (III).

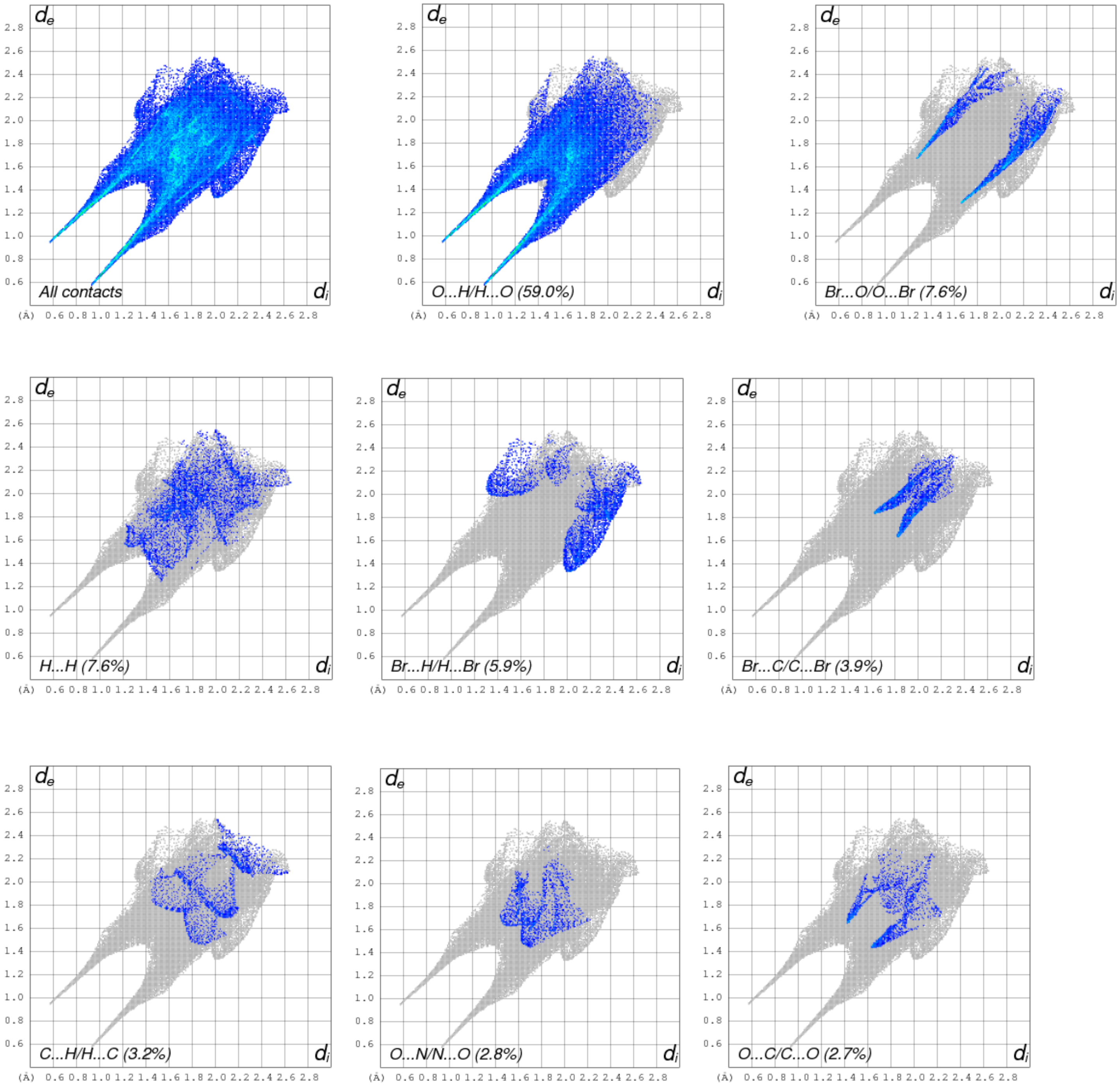
All contacts
O...H/H...O (59.0%)
Br...O/O...Br (7.6%)
H...H (7.6%)
Br...H/H...Br (5.9%)
Br...C/C...Br (3.9%)
C...H/H...C (3.2%)
O...N/N...O (2.8%)
O...C/C...O (2.7%)

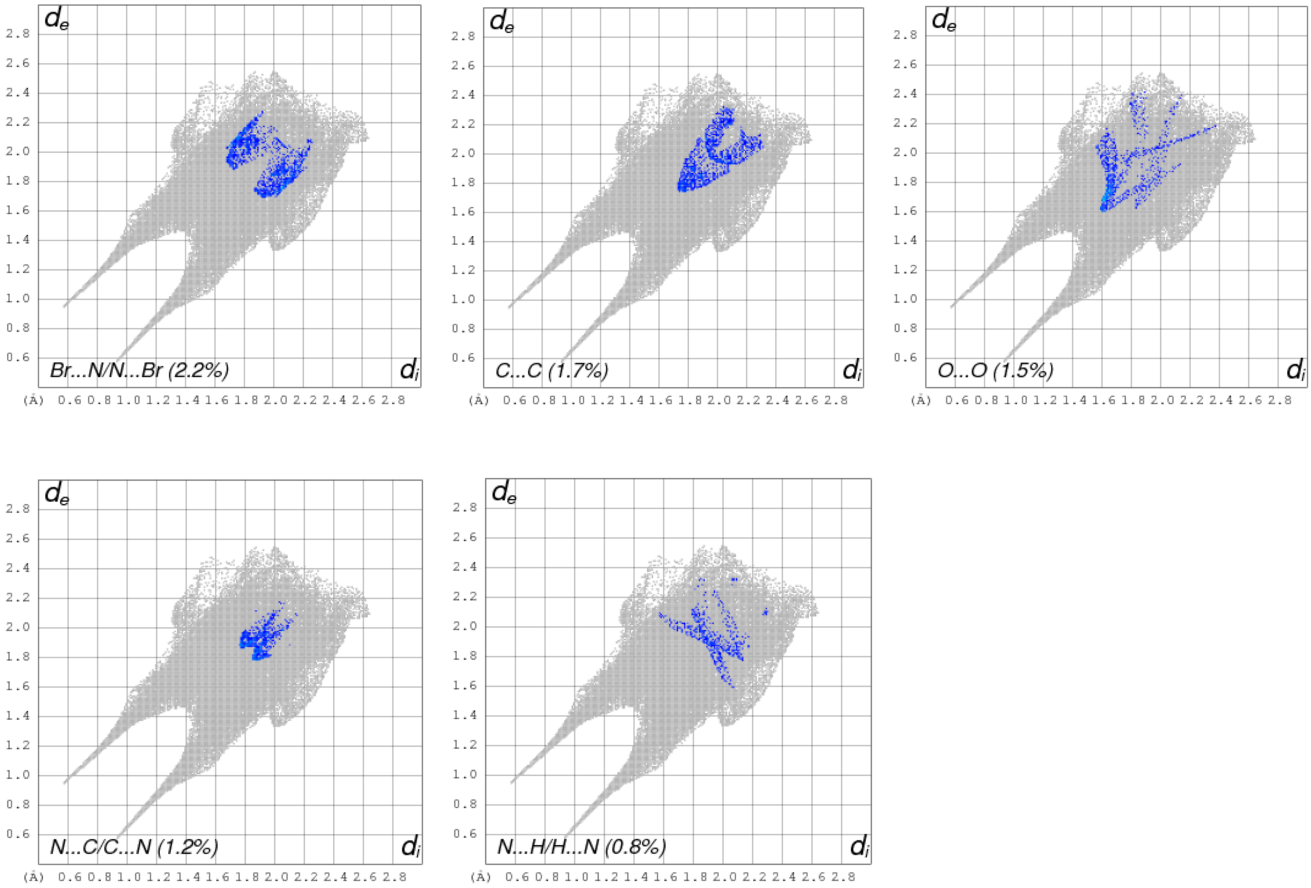

Figures S4: decomposed two-dimensional fingerprint plots for compound (IV).

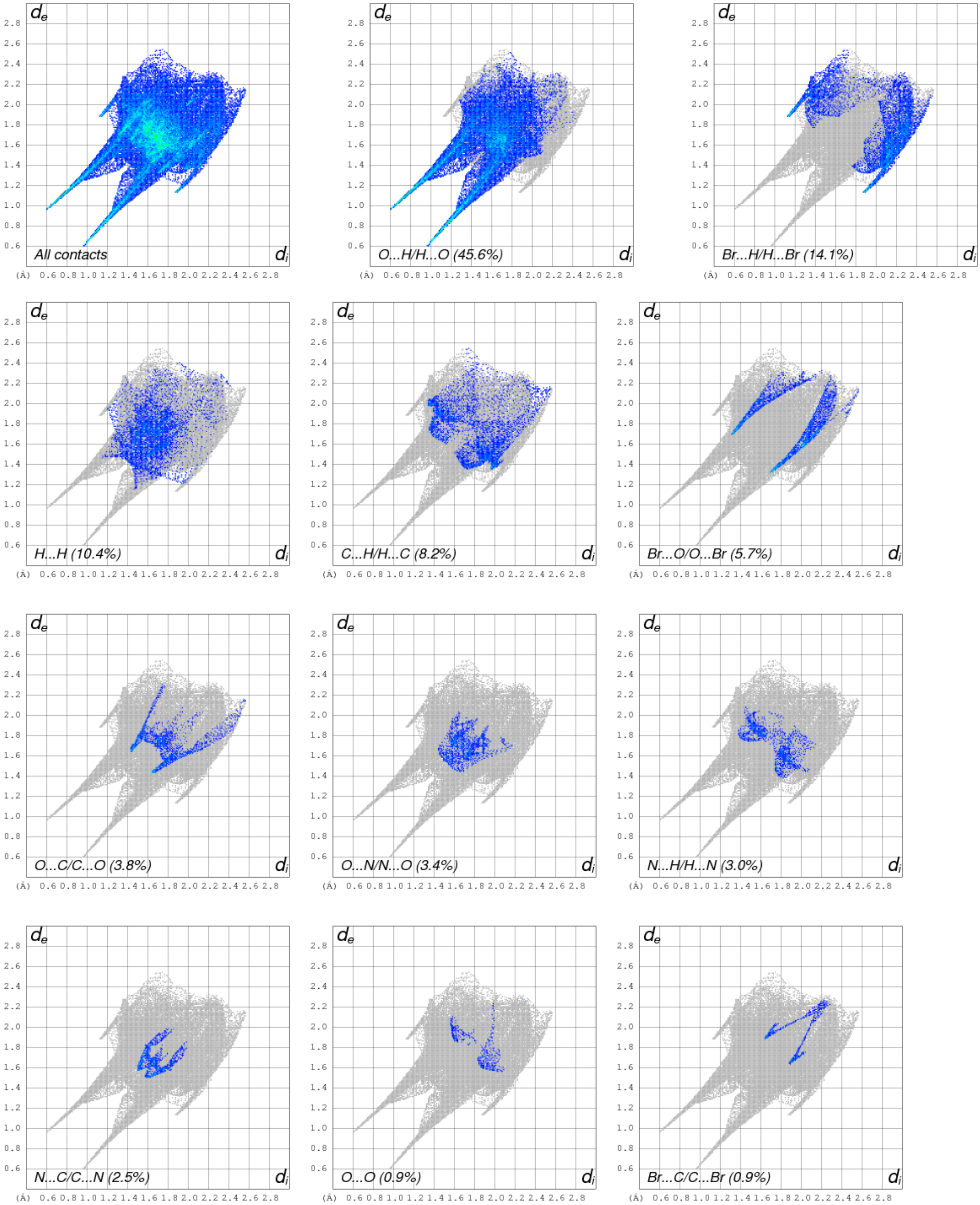
All contacts
O...H/H...O (45.6%)
Br...H/H...Br (14.1%)
H...H (10.4%)
C...H/H...C (8.2%)
Br...O/O...Br (5.7%)
O...C/C...O (3.8%)
O...N/N...O (3.4%)
N...H/H...N (3.0%)
N...C/C...N (2.5%)
O...O (0.9%)
Br...C/C...Br (0.9%)
d_e
d_i
(Å) 0.6 0.8 1.0 1.2 1.4 1.6 1.8 2.0 2.2 2.4 2.6 2.8

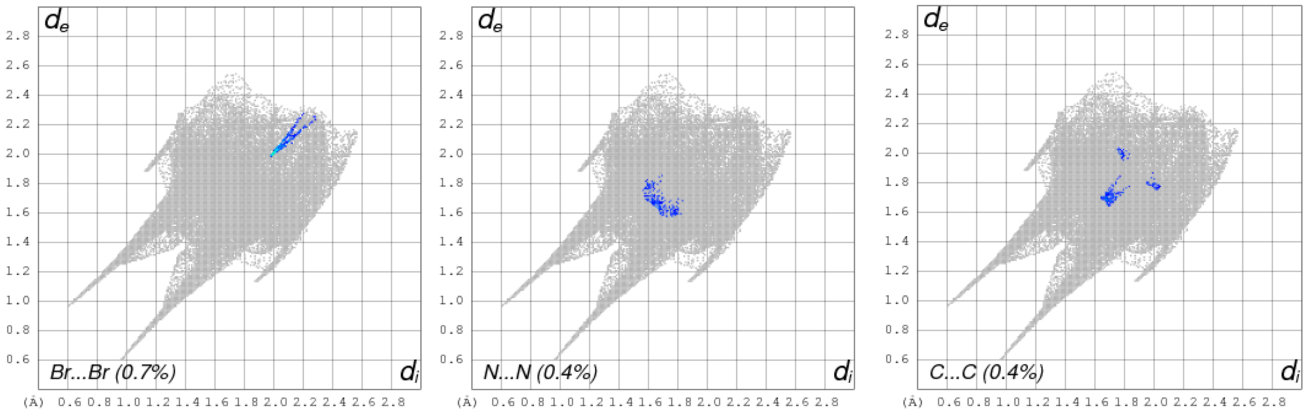

Figures S5: decomposed two-dimensional fingerprint plots for compound (V).

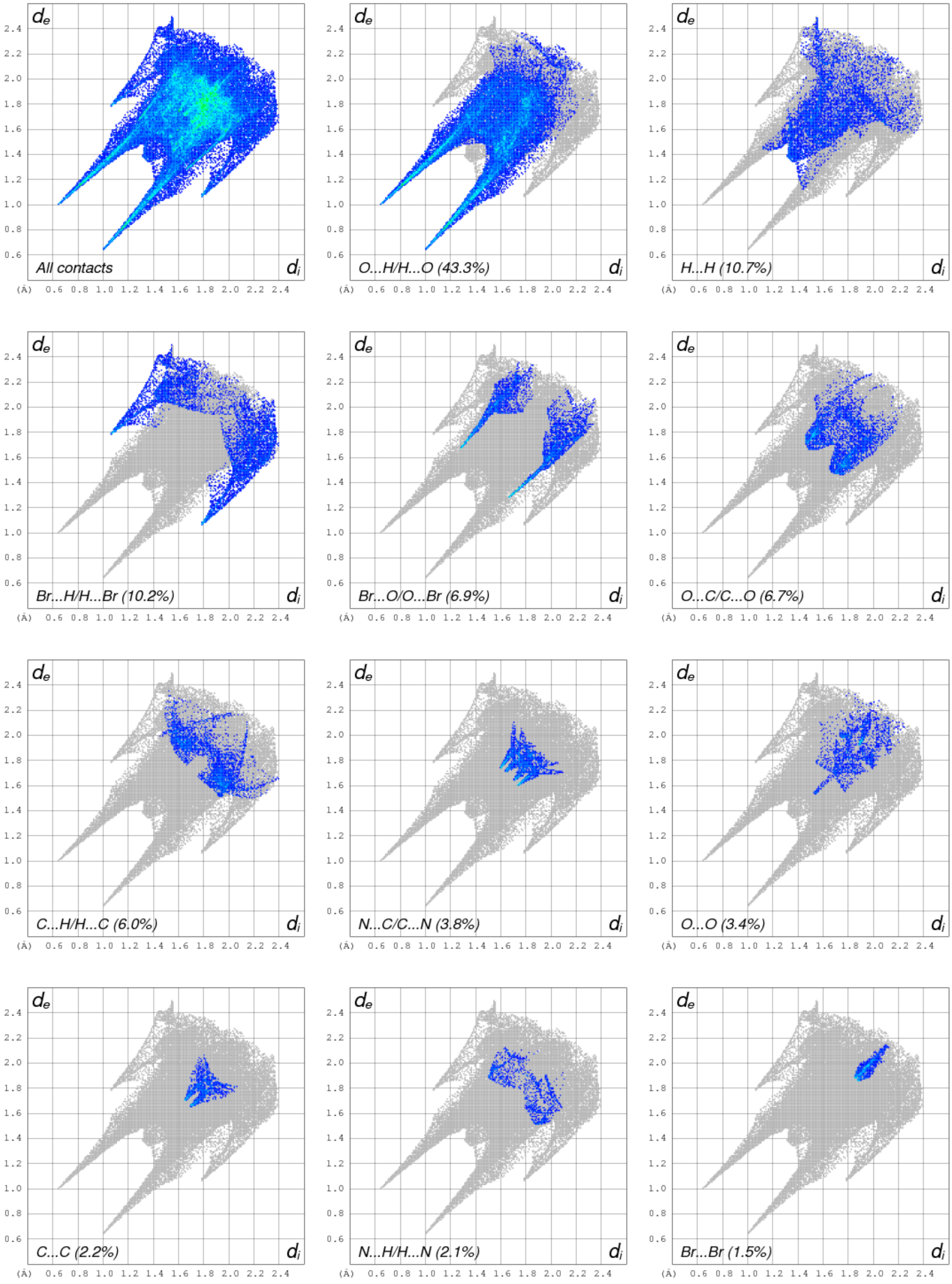

de
di
(Å)
All contacts
O...H/H...O (43.3%)
H...H (10.7%)
Br...H/H...Br (10.2%)
Br...O/O...Br (6.9%)
O...C/C...O (6.7%)
C...H/H...C (6.0%)
N...C/C...N (3.8%)
O...O (3.4%)
C...C (2.2%)
N...H/H...N (2.1%)
Br...Br (1.5%)

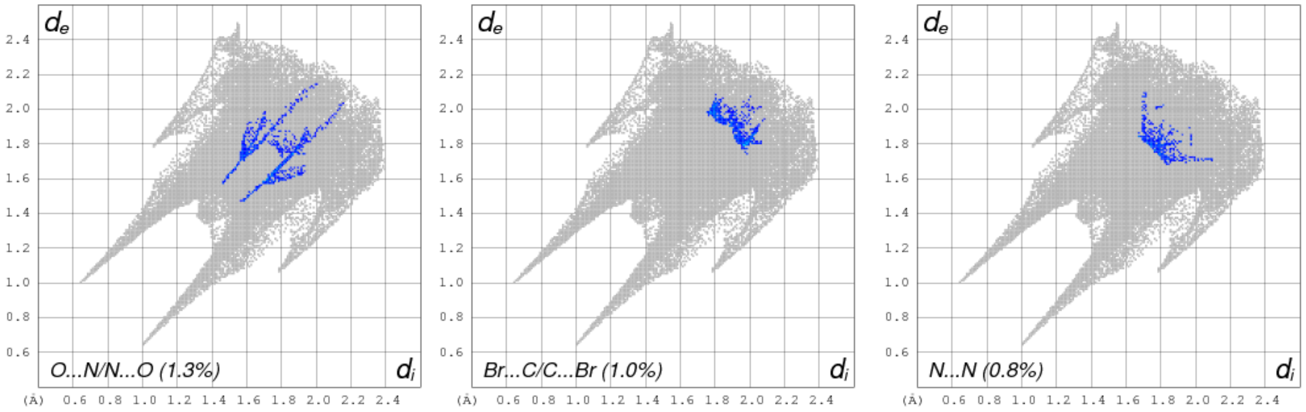

Figure 6. decomposed two-dimensional fingerprint plots for compound (VI).

Table S1. List of Bond-critical-points for the HBs emerged from the structural analysis (see Table 1), corresponding geometrical parameters (Å,º) and details of the topological analysis at the bond-CP for compound (I); ρ = electron density; $\nabla^2\rho$ = Laplacian of the electron density.

HB	*Atomic Index (see indicated cartesian coordinates)*	*Index of Bond- CP* [c]	*D*—H	H···*A*	*D*···*A*	*D*—H···*A*	ρ	$\nabla^2\rho$
N2—H2A···Cl1	40-42-48 [a]	151	0.98	2.46	3.34	148	0.015	0.043
N2—H2B···Cl1C	40-41-49 [a]	143	0.98	2.23	3.16	156	0.021	0.067
N1—H1···O1B	33-34-29 [a]	97	0.98	1.79	2.76	167	0.034	0.11
N3—H3···Cl1	27-38-48 [a]	150	0.98	2.24	3.17	158	0.024	0.059
N2A—H2A1···Cl1A	2-3-15 [a]	73	1.00	2.24	3.12	147	0.023	0.064
N2A—H2A2···O1A [i]	19-69-50 [b]	133	0.99	2.46	3.38	155	0.0091	0.028
N1A—H1A···Cl1 [ii]	13-70-10 [b]	89	0.99	2.52	3.37	144	0.024	0.038
N3A—H3A···Cl1A	13-14-15 [a]	82	1.04	2.05	3.03	156	0.034	0.079
N2B—H2B1···O2$_{W}$	19-20-30 [a]	67	1.00	1.00	2.83	152	0.027	0.082
N2B—H2B2···Cl1A	19-21-15 [a]	75	1.01	2.19	3.13	156	0.023	0.072
N1B—H1B···O1	26-27-36 [a]	130	1.01	1.79	2.77	163	0.034	0.11
N3B—H3B···Cl1B	16-17-47 [a]	69	1.06	1.88	2.92	171	0.050	0.096
N2C—H2C1···Cl1C	61-63-49 [a]	146	1.02	2.14	3.06	149	0.028	0.074
N1C—H1C···Cl1B [iii]	13-70-10 [b]	89	0.99	2.52	3.37	145	0.014	0.038
N3C—H3C···Cl1C	51-52-49 [a]	134	1.04	2.097	3.05	152	0.031	0.075
O2$_{W}$—H2A···Cl1B	30-31-47 [a]	66	0.96	2.16	2.97	142	0.026	0.076
O2$_{W}$—H2B···Cl1 [iv]	11-79-10 [b]	84	0.99	2.18	3.16	170	0.026	0.060

[a] See Coordinates in Cluster-Compund-I-1. [b] See Coordinates in Cluster-Compund-I-2 [c] For the coordinates of the critical point see atoms indicated with CP in the corresponding Cluster-Compund indicated in the first column.

Table S2. List of Bond-critical-points for the Halogen Bonds as emerged from the structural analysis, corresponding geometrical parameters (Å,º) and details of the topological analysis at the bond-CP for compound (I); ρ = electron density; $\nabla^2\rho$ = Laplacian of the electron density.

Atomic index (see indicated cartesian coordinates)	*Index of Bond- CP* [c]	*X*—Br	Br···*O*	*C*—Br···*O*	ρ	$\nabla^2\rho$
74-23-12 [a]	119	1.84	3.18	175	0.015	0.043
43-44-54 [a]	99	1.83	2.97	166	0.011	0.040

[a] See Coordinates in Cluster-Compund-I-1.

Table S3. List of Bond-critical-points for the HBs emerged from the structural analysis (see Table 2), corresponding geometrical parameters (Å,º) and details of the topological analysis at the bond-CP for compound (II); ρ = electron density; $\nabla^2\rho$ = Laplacian of the electron density.

HB	*Atomic index (see indicated cartesian coordinates)*	*Index of Bond- CP* [c]	*D*—H	H···*A*	*D*···*A*	*D*—H···*A*	ρ	$\nabla^2\rho$
N2—H2A···O2$_W$^{i}	32-34-5[a]	54	1.00	1.90	2.83	155	0.072	0.074
N2—H2B···O2$_W$	32-33-3[a]	50	1.00	1.81	2.78	162	0.033	0.094
N1—H1···Br2	29-30-7[a]	95	1.01	2.20	3.21	174	0.030	0.052
N3—H3···O1^{ii}	25-26-40[a]	66	0.99	1.74	2.72	172	0.035	0.099
O2$_W$—H2B···Br2^{iv}	3-38-2[b]	41	0.94	2.25	3.20	172	0.013	0.035
O2$_W$—H2A···Br2^{iii}	8-22-7[a]	96	0.94	2.51	3.41	154	0.014	0.035

[a] See Coordinates in Cluster-Compund-II-1. [b] See Coordinates in Cluster-Compund-II-2 [c] For the coordinates of the critical point see atoms indicated with CP in the corresponding Cluster-Compund indicated in the first column.

Table S4. List of additional interactions as emerged from the structural analysis, corresponding geometrical parameters (Å,º) and details of the topological analysis at the bond-CP for compound (II); ρ = electron density; $\nabla^2\rho$ = Laplacian of the electron density.

Atomic Index (see indicated cartesian coordinates)	*Index of Bond- CP* [c]	*X*—Br	Br···*Br*	*C*—Br···*Br*	ρ	$\nabla^2\rho$
27-39-6[a]	73	1.85	3.43	160	0.010	0.028

[a] See Coordinates in Cluster-Compund-II-1.

Table S5. List of Bond-critical-points for the HBs emerged from the structural analysis (see Table 3), corresponding geometrical parameters (Å,º) and details of the topological analysis at the bond-CP for compound (III); ρ = electron density; $\nabla^2\rho$ = Laplacian of the electron density.

HB	*Atomic index (see indicated cartesian coordinates)*	*Index of Bond- CP* [c]	*D*—H	H···*A*	*D*···*A*	*D*—H···*A*	ρ	$\nabla^2\rho$
N2—H2A···O3	87-89-55[a]	173	0.96	1.88	2.83	173	0.029	0.090
N2—H2B···O5$_w$^{i}	87-88-15[b]	45	0.98	1.88	2.84	171	0.026	0.080
N1—H1···O4C^{ii}	95-95-97[a]	109	0.97	2.28	3.04	170	0.030	0.081
N3—H3···O2	84-85-57[a]	160	0.98	1.83	2.81	174	0.033	0.098
C6—H6···O1C^{ii}	44-45-39[a]	220	1.01	2.44	3.0	150	0.0094	0.036
N2C—H2CA···O3	29-31-55[a]	194	0.96	2.02	2.95	163	0.022	0.065
N2C—H2CB···O3C	29-30-34[a]	211	0.96	2.10	3.04	169	0.019	0.051
N1C—H1C···O3C^i	42-43-38[a]	218	0.98	2.35	3.05	138	0.012	0.041
N1C—H1C···O4C^i	42-43-39[a]	219	0.98	2.34	3.31	172	0.012	0.035
N3C—H3C···O2C	84-85-57[a]	114	0.98	1.83	2.81	173	0.032	0.094
O5$_w$—H52···O1	15-17-10[a]	154	0.92	1.91	2.83	170	0.024	0.087
N2A—H2AA···O3A	49-50-64[a]	170	0.97	1.99	2.96	173	0.022	0.068
N2A—H2AB···O6$_w$^{iii}	87-88-15[a]	164	0.96	1.77	2.72	171	0.029	0.090
N1A—H1A···O2B^{iv}	42-43-39[a]	218	0.98	2.30	3.05	130	0.012	0.035
N1A—H1A···O4B^{iv}	42-43-38[a]	219	0.98	2.30	3.31	172	0.012	0.041
N3A—H3A···O2A	52-53-62[a]	179	0.97	1.97	2.94	176	0.023	0.070
N2B—H2BA···O3A	69-70-64[a]	139	0.96	1.94	2.88	165	0.026	0.077
N2B—H2BB···O3B	29-30-34[a]	211	0.96	2.10	3.04	169	0.019	0.051
N1B—H1B···O4B	94-95-97	109	0.97	2.55	3.51	168	0.013	0.043
N3B—H3B···O2B	26-27-33[a]	226	0.97	1.87	2.82	168	0.032	0.084
O6$_w$—H61···O1A	65-66-57[a]	169	0.92	1.95	2.87	173	0.022	0.079
O6$_w$—H62···O2A	65-67-83[a]	135	0.95	1.92	2.79	152	0.025	0.084

[a] See Coordinates in Cluster-Compund-III-1. [b] See Coordinates in Cluster-Compund-III-2 [c] For the coordinates of the critical point see atoms indicated with CP in the corresponding Cluster-Compund indicated in the first column.

Table S6. List of Bond-critical-points for Halogen Bonds as emerged from the structural analysis, corresponding geometrical parameters (Å,º) and details of the topological analysis at the bond-CP for compound (III); = electron density; ∇^2 = Laplacian of the electron density.

Atomic index (see indicated cartesian coordinates)	*Index of Bond- CP* [d]	*X*—Br	Br···*O*	*C*—Br···*O*	ρ	$\nabla^2\rho$
91-10 [a]	132	1.86	2.90	158	0.014	0.056
19-7 [c]	114	1.86	3.07	155	0.010	0.039
18-15 [a]	180	1.84	3.00	160	0.009	0.037
1-2 [b]	41	1.85	2.92	164	0.012	0.051

[a] See Coordinates in Cluster-Compund-III-1. [b] See Coordinates in Cluster-Compund-III-2 [c] See Coordinates in Cluster-Compund-III-3 [d] For the coordinates of the critical point see atoms indicated with CP in the corresponding Cluster-Compund indicated in the first column.

Table S7. Interlayer Bond-CP interactions for compound (III), corresponding geometrical parameters (Å,º) and details of the topological analysis; ρ = electron density; $\nabla^2\rho$ = Laplacian of the electron density.

Atomic index (see indicated cartesian coordinates)	*Index of Bond- CP* [b]	*X*—Y	ρ	$\nabla^2\rho$
52-56 [a]	190	3.10	0.0074	0.025
36-33 [a]	226	3.10	0.031	0.084

[a] See Coordinates in Cluster-Compund-III-1. [b] For the coordinates of the critical point see atoms indicated with CP in the corresponding Cluster-Compund indicated in the first column

Table S8. List of Bond-critical-points for the HBs emerged from the structural analysis (see Table 4), corresponding geometrical parameters (Å,º) and details of the topological analysis at the bond-CP for compound (IV); ρ = electron density; $\nabla^2\rho$ = Laplacian of the electron density.

HB	*Atomic index (see indicated cartesian coordinates)*	*Index of Bond- CP* [c]	*D*—H	H···*A*	*D*···*A*	*D*—H···*A*	ρ	$\nabla^2\rho$
N2—H2A···O2	29-31-44 [a]	63	1.02	2.07	3.03	153	0.029	0.090
N2—H2B···$O4^{i}$	29-30-27 [a]	73	1.00	1.97	2.87	148	0.026	0.080
N1—H1···$O4^{ii}$	37-38-52 [a]	107	1.01	2.08	3.02	154	0.030	0.081
N3—H3···O3	41-42-45 [a]	68	1.02	2.01	3.02	173	0.033	0.098
$O6_w$—H6A···$O2^{i}$	4-23-20 [b]	35	0.98	1.58	2.48	173	0.0094	0.036
$O6_w$—H6B···$O3^{iii}$	20-21-19 [a]	119	1.04	1.51	2.54	172	0.022	0.065
$O6_w$—H6C···$O5^{iv}$	56-54-49 [a]	128	1.08	1.34	2.43	177	0.019	0.051

[a] See Coordinates in Cluster-Compund-IV-1. [b] See Coordinates in Cluster-Compund-IV-2 [c] For the coordinates of the critical point see atoms indicated with CP in the corresponding Cluster-Compund indicated in the first column

Table S9. List of Bond-critical-points for Halogen Bonds as emerged from the structural analysis, corresponding geometrical parameters (Å,º) and details of the topological analysis at the bond-CP for compound (IV); ρ = electron density; $\nabla^2\rho$ = Laplacian of the electron density.

Atomic index (see indicated cartesian coordinates)	*Index of Bond- CP* [b]	*X*—Br	Br···*O*	*C*—Br···*O*	ρ	$\nabla^2\rho$
34-10 [a]		1.86	3.08	175	0.0089	0.033

[a] See Coordinates in Cluster-Compund-IV-1.[b] For the coordinates of the critical point see atoms indicated with CP in the corresponding Cluster-Compund indicated in the first column.

Table S10. List of Bond-critical-points for the HBs emerged from the inter-chain (SO_4^{2-}/H_3O^+) HBs in compound (IV). See coordinates on Compounds IV-3.

Atomic index (see indicated cartesian coordinates)	*Index of Bond- CP*	*D*—H	H···*A*	*D*···*A*	*D*—H···*A*	ρ	∇²ρ
4-17-8	28	0.97	1.56	2.53	173	0.056	0.016
4-18-15	40	0.98	1.45	2.43	174	0.087	0.015
4-19-20	30	1.01	1.01	2.53	174	0.074	0.018

Table S11. List of Bond-critical-points for the HBs emerged from the structural analysis (see Table 5), corresponding geometrical parameters (Å,º) and details of the topological analysis at the bond-CP for compound (V); = electron density; ∇² = Laplacian of the electron density.

HB	*Atomic index (see indicated cartesian coordinates)*	*Index of Bond- CP* [c]	*D*—H	H···*A*	*D*···*A*	*D*—H···*A*	ρ	∇²ρ
N2—H2A···O3	40-41-18 [a]	125	1.00	1.71	3.03	177	0.042	0.13
N2—H2B···O1^{i}	1-35-2 [b]	47	0.98	2.19	2.87	117	0.014	0.098
N1—H1···O3^{ii}	1-2-28 [a]	121	1.01	1.73	2.74	171	0.040	0.11
N3—H3···O2	11-12-70 [a]	150	0.99	1.78	2.77	175	0.036	0.11
O4—H4···O2^{iii}	58-59-65 [a]	153	0.94	1.78	2.71	170	0.034	0.11
O5—H5···O4^{iv}	67-68-60 [a]	164	0.94	1.75	2.68	171	0.036	0.12

[a] See Coordinates in Cluster-Compund-V-1

[b] See Coordinates in Cluster-Compund-V-2

[c] For the coordinates of the critical point see atoms indicated with CP in the corresponding Cluster-Compund indicated in the first column

Table S12. List of Bond-critical-points for Halogen Bonds as emerged from the structural analysis, corresponding geometrical parameters (Å,º) and details of the topological analysis at the bond-CP for compound (V); ρ = electron density; ∇²ρ = Laplacian of the electron density.

Atomic index (see indicated cartesian coordinates)	*Index of Bond- CP* [b]	*X*—Br	Br···*O*	*C*—Br···*O*	ρ	∇²ρ
1-5 [a]		1.86	3.09	171	0.094	0.032

[a] See Coordinates in Cluster-Compund-V-1. [b] For the coordinates of the critical point see atoms indicated with CP in the corresponding Cluster-Compund indicated in the first column

Table S13. List of Bond-critical-points for the HBs emerged from the structural analysis (see Table 6), corresponding geometrical parameters (Å,º) and details of the topological analysis at the bond-CP for compound (VI); ρ = electron density; ∇²ρ = Laplacian of the electron density.

HB	*Atomic index (see indicated cartesian coordinates)*	*Index of Bond- CP* [c]	*D*—H	H···*A*	*D*···*A*	*D*—H···*A*	ρ	∇²ρ
N2—H21···O2^{i}	10-11-23 [a]	60	1.01	1.89	2.89	171	0.030	0.077
N2—H22···O6$_{w}^{ii}$	10-12-18 [a]	54	0.99	2.52	3.48	162	0.070	0.027
N1—H1···O2	3-4-30 [a]	113	1.00	1.88	2.87	168	0.039	0.079
N3—H3···O3^{i}	13-14-22 [a]	85	1.00	1.83	2.83	174	0.034	0.088
O5—H5···O6$_{w}$	13-14-22 [b]	60	0.97	1.67	2.63	167	0.043	0.10
O6$_{W}$—H61···O1^{iii}	15-16-22 [a]	109	0.94	1.97	2.80	146	0.021	0.078
O6$_{W}$—H62···O3^{iv}	35-36-29 [a]	120	0.95	1.93	2.80	152	0.026	0.081

[a] See Coordinates in Cluster-Compund-VI-1. [b] See Coordinates in Cluster-Compund-VI-2 [c] For the coordinates of the critical point see atoms indicated with CP in the corresponding Cluster-Compund indicated in the first column

Table S14. List of Bond-critical-points for Halogen Bonds as emerged from the structural analysis, corresponding geometrical parameters (Å,º) and details of the topological analysis at the bond-CP for compound (VI); ρ = electron density; $\nabla^2\rho$ = Laplacian of the electron density.

Atomic index (see indicated cartesian coordinates)	*Index of Bond- CP* [b]	*X*—Br	Br···*O*	*C*—Br···*O*	ρ	$\nabla^2\rho$
39-32 [a]	65	1.86	3.02	166	0.011	0.035

[a] See Coordinates in Cluster-Compund-V-1. [b] For the coordinates of the critical point see atoms indicated with CP in the corresponding Cluster-Compund indicated in the first column

Table 15. Cartesian coordinates, coordinates and index of the bond CP for all the cluster adopted in this work (see Tables S1-S14).

Cluster Compound I-1

C	-10.9038181	-0.103156477	0.234030753
N	-10.9117632	-1.36685801	0.462537944
H	-10.0721884	-1.90505672	0.336937606
H	-11.7465496	-1.81260037	0.763848245
C	-12.0295029	0.734283984	0.359329402
Br	-13.6262341	3.11698392E-02	0.953261435
C	-11.8992300	2.00316596	2.17875261E-02
H	-12.7574692	2.63636613	7.68871009E-02
N	-10.7546206	2.52640486	-0.381634682
H	-10.7292089	3.49334836	-0.609451056
C	-9.59259796	1.79599535	-0.500830054
O	-8.55300999	2.22788119	-0.859184623
N	-9.76747990	0.483644366	-0.172415093
H	-8.93531036	-0.141272768	-0.248301804
Cl	-7.91799498	-1.91802275	-0.262032479
N	-2.56175089	-1.72640944	-0.460160792
H	-2.35510492	-2.76212239	-0.409128428
C	-3.81478572	-1.26543689	-0.472816437
N	-4.79191780	-2.09458566	-0.376671284
H	-4.61049652	-3.08111668	-0.292267859
H	-5.74097347	-1.75746632	-0.385489643
C	-3.98753595	0.146468073	-0.589968860
Br	-5.67045689	0.898352385	-0.595008969
C	-2.88767195	0.891750932	-0.688414454
H	-2.98377275	1.94754088	-0.807869256
N	-1.67270136	0.354145885	-0.643339932
H	-0.875147939	0.973715425	-0.687372863
C	-1.43790114	-0.972249210	-0.541510105
O	-0.343694299	-1.45805252	-0.520052791
O	-4.47977066	-4.69246101	0.715813994
H	-3.58015752	-5.00313330	0.584422886

H	-5.01539183	-5.36683989	1.10193551
N	1.86749458	7.92336985E-02	7.90646672E-02
H	1.08128989	-0.505242169	2.34073307E-03
C	1.66827643	1.39699423	-4.55913395E-02
O	0.602686465	1.89002001	-0.262771457
N	2.78881836	2.14332438	0.124472290
H	2.68431687	3.11969519	0.161834896
C	4.01957750	1.64691651	0.244005710
N	5.00468111	2.47996259	0.292511642
H	5.92546463	2.14838719	0.381009787
H	4.83584261	3.44661450	0.240624368
C	4.17017841	0.261332929	0.293466806
Br	5.79841757	-0.543376148	0.441202492
C	3.05527377	-0.462800205	0.218991175
H	3.12516165	-1.44156539	0.271446407
Cl	-1.77335978	-4.54290533	-0.510119140
Cl	3.22125673	5.28021717	-8.60532448E-02
Cl	8.14796829	2.16599846	0.539164543
C	10.9465408	8.62646848E-02	0.157271445
N	9.72398376	-0.427416593	0.184280768
H	8.94285583	0.233629897	0.364347845
C	9.38821507	-1.74015570	-4.66720783E-04
O	8.27389145	-2.13278747	-1.49850990E-03
N	10.4873610	-2.55148697	-0.175592020
H	10.3388796	-3.52726102	-0.290696591
C	11.7444277	-2.09699988	-0.197908163
H	12.5433836	-2.77726507	-0.391630262
C	12.0195131	-0.813941002	1.44401966E-02
Br	13.7512531	-0.184827909	-3.67258005E-02
N	11.0912561	1.36156750	0.259746879
H	11.9996376	1.76259696	0.241212010
H	10.2687063	1.96493983	0.360568553

Coordinates and Index of Bond-Critical Points

-2.99386859	-4.84014606	0.214455560	66
-4.59172297	-3.65671396	8.99989307E-02	67
-2.17910695	-3.30476046	-0.440034896	69
-9.35637665	-1.88730311	0.135041490	73
-6.46226406	-1.82222176	-0.339574814	75
-8.61754227	-0.707358062	-0.251511812	82
-12.7816954	0.402237117	0.638229966	85
-6.74379015	-0.582299948	-0.419195920	86

0.565874219	-0.819150031	-0.195114627	97
7.11189890	-1.35061562	0.163133472	99
-4.78450966	0.499582052	-0.592511296	109
4.95090389	-0.119664460	0.365896076	114
-7.19488001	1.54862094	-0.741744518	119
12.8349257	-0.515478015	-1.00280913E-02	123
-0.339574426	1.27133250	-0.521597087	130
6.95792484	0.857454896	0.480147302	132
8.69542027	0.856335104	0.418497384	134
6.66888523	2.16397905	0.430844009	143
9.57512569	2.01359510	0.416971296	146
2.86916232	3.82520342	7.85132200E-02	150
4.26033974	4.04903460	0.127343625	151

Cluster Compound I-2

C	-8.82845020	-3.73910809	-5.62907532E-02
N	-9.98403740	-3.10145044	-0.169312820
C	-10.1001921	-1.75564694	-7.17237070E-02
N	-8.89906216	-1.14403975	0.137874946
C	-7.71554995	-1.73704994	0.249742299
C	-7.70256042	-3.12908483	0.171072021
O	-11.1113148	-1.15283465	-0.168649420
N	-6.66477585	-1.00790834	0.401817501
Br	-6.11563635	-4.03405476	0.359521866
Cl	-3.89751744	-6.25733662	0.667999566
O	-0.994358599	-6.74067593	1.64466667
C	-1.11163747	-4.03993797	-4.24891412E-02
N	-2.25709462	-3.41536283	0.186855853
C	-2.37577152	-2.06630182	0.199192792
N	-1.18773603	-1.43778527	-3.37153375E-02
C	-1.42343808E-02	-2.01786780	-0.260155529
C	2.36953594E-04	-3.41181612	-0.288756907
O	-3.37804508	-1.47491717	0.401329845
N	1.02736962	-1.27688468	-0.417969137
Br	1.57121956	-4.29620028	-0.639026523
Cl	-0.391194612	1.46364796	-4.47531901E-02
N	-2.12413001	4.07612848	-0.312770307
C	-3.37059212	4.36013269	-0.383718073
N	-3.74149704	5.62401628	-0.526548684
C	-5.01824427	6.07159185	-0.626971960
N	-5.93700552	5.10491657	-0.568904400
C	-5.65115976	3.81977844	-0.401988477

C	-4.39918852	3.38825965	-0.322598994
O	-5.27544737	7.22600555	-0.757682920
Br	-4.00603199	1.61704540	-0.150236353
O	-0.126710609	5.79690456	-1.29289436
Cl	-1.92175877	8.03777218	-0.410846740
N	1.82497573	4.74575758	0.971341550
C	2.74774146	5.70726252	1.04859710
N	4.01531744	5.27152777	0.839131534
C	4.37429094	4.02305365	0.578700066
C	3.34169006	3.05635190	0.504496098
C	2.09756708	3.47773576	0.690338671
O	2.50131011	6.84776545	1.28190529
N	5.61374521	3.74809813	0.412833542
Br	3.71998620	1.30432737	0.174674109
Cl	5.83049202	7.69117880	0.800566256
Cl	7.32193375	1.16757560	-0.155446544
N	6.51958609	-1.72635984	-0.340627640
C	5.31845665	-2.33796692	-0.550227284
N	5.43461084	-3.68377018	-0.647816420
C	6.59019899	-4.32142830	-0.534793377
C	7.71608829	-3.71140480	-0.307430595
C	7.70309877	-2.31937003	-0.228760317
O	4.30733347	-1.73515463	-0.647153020
N	8.75387287	-1.59022832	-7.66850933E-02
Br	9.30301094	-4.61637545	-0.118980728
H	8.67257214	-0.621317089	-4.50388007E-02
H	9.62858868	-2.00764465	7.18642073E-03
H	4.62966394	-4.20615482	-0.807739675
H	6.51760244	-0.756029606	-0.270946085
H	6.59664679	-5.28994226	-0.626119316
H	6.28261232	4.45144176	0.478611380
H	5.88773918	2.83459044	0.220921665
H	0.897308409	4.99115419	1.13138294
H	4.66906595	5.99108601	0.874488652
H	1.36541724	2.84127712	0.617698133
H	-1.45872855	4.78394604	-0.364107460
H	-1.84134495	3.15126705	-0.207582474
H	-6.87194777	5.35970736	-0.654717088
H	-3.08935618	6.34564877	-0.545476973
H	-6.37917757	3.17798376	-0.334986359
H	0.946529031	-0.308566928	-0.370790422
H	1.89466465	-1.68471646	-0.584922075

H	-3.05237079	-3.95023513	0.353731871
H	-1.19164109	-0.464973778	-2.89705936E-02
H	-1.10199356	-5.01257801	-2.58154292E-02
H	-6.74607611	-3.89970243E-02	0.433462828
H	-5.79005909	-1.42532480	0.485688031
H	-10.7889843	-3.62383485	-0.329236120
H	-8.90104580	-0.173709676	0.207556531
H	-8.82200241	-4.70762110	-0.147616729
H	-0.690045774	-7.66314363	1.59130204
H	-1.93761408	-6.67311430	1.41640377
H	-0.431560308	6.72006750	-1.25770748
H	0.801266909	5.74907160	-1.58093834

Coordinates and Index of Bond-Critical Points

-2.55166960	-6.53612947	1.16981447	84
-1.01596332	-5.68316603	0.647734940	86
-5.05620575	-5.12078524	0.508411288	87
-3.30805659	-4.74754286	0.455213487	89
-6.92042923	-3.57021070	0.262688428	93
0.734074652	-3.82153821	-0.450822920	100
3.50922608	-4.07099867	-0.684339046	102
-4.71281862	-2.57815313	0.381235391	106
8.46772003	-4.13908195	-0.219116092	108
3.01446629	-2.87609267	-0.640274286	119
-4.86606169	-1.46457255	0.433788627	122
2.83324695	-1.73266375	-0.593561172	133
-3.63830996	2.40634698E-02	0.149832547	140
-5.67678404	0.464849919	0.163263723	143
-0.916035712	0.138061538	-3.53162698E-02	147
4.07014036	-0.270628244	-0.270842791	149
0.466497421	0.250637591	-0.257449895	151
2.01707125	0.201552987	-0.125616059	152
6.79242373	-0.159232229	-0.234361455	154
8.19252968	-5.86449429E-02	-8.62605944E-02	156
-2.18040252	1.63009834	-0.105651870	157
5.54945326	1.31181097	2.29166206E-02	159
-4.21229219	2.55076718	-0.240480542	160
0.753769100	2.36663914	0.381406784	161
-1.34995008	2.60776186	-0.157313809	163
3.52102542	2.23334265	0.348858029	164
6.37593031	2.29570293	0.101544626	166
-0.970195413	5.10914755	-0.724772036	187

0.775768936	5.28513241	-9.84050259E-02	190
-2.72593236	6.86747789	-0.499488115	197
5.03235674	6.51583624	0.847197592	199
-0.922646284	7.11523438	-0.968295753	200

Cluster Compound II-1

C	-2.54300523	-1.06742203	-2.72599813E-02
Br	-2.34650922	-4.99265194	-8.90006274E-02
O	0.682407916	-3.50032759	0.419962674
C	1.98941839	-1.15587991E-02	4.95685451E-02
O	4.57327461	-2.87516904	0.386109442
Br	7.59437180	-1.38147831	-0.163685739
Br	6.77052438E-02	5.55897713	-7.35200420E-02
O	-2.95177674	4.06226969	0.476944089
N	-3.50523734	-1.99721193	-8.58693644E-02
C	-4.78117704	-1.69256616	-7.24684596E-02
C	-5.21295404	-0.434528500	-5.11568524E-02
C	-4.25333500	0.590222299	-3.79560776E-02
N	-2.98740673	0.214858025	-1.87870692E-02
Br	-7.01775026	-3.19855250E-02	-4.62564193E-02
H	-5.44402266	-2.51215529	-8.99294689E-02
H	-3.18949294	-2.97146368	-9.71696004E-02
H	-2.25964522	0.903240681	-2.58017872E-02
N	-4.53168726	1.83992636	-3.72342691E-02
H	-5.47109365	2.11550617	-4.76992577E-02
H	-3.84493589	2.56917882	2.54370272E-02
H	-2.79112816	3.98029017	1.39015138
H	-2.12780285	4.29501343	7.69672319E-02
C	0.290049374	1.65288663	-4.47126366E-02
O	-0.872611403	1.89418530	-7.60197863E-02
N	0.723490596	0.376839459	9.16009117E-03
H	1.07425703E-02	-0.309099138	2.43642344E-03
C	2.96965623	1.01413321	3.53265442E-02
C	2.53450084	2.25837111	-1.38871446E-02
N	1.23619115	2.57370687	-4.41630073E-02
H	0.931662202	3.53820896	-7.70525187E-02
H	3.19236183	3.08073878	-3.65566798E-02
N	2.27113032	-1.24967360	7.15355948E-02
H	1.57892990	-1.96722496	6.67805895E-02
H	3.21107745	-1.58556044	0.105132826
H	5.41988897	-2.61603761	5.08706719E-02

H	4.67816877	-3.00804925	1.30071688
H	0.537474930	-3.42459559	1.33601511
H	-0.144473240	-3.72907281	3.43858898E-02
Br	4.76127291	0.545673251	2.80581340E-02
O	-1.40131640	-1.32770264	1.20363040E-02

Coordinates and Index of Bond-Critical Points

-0.862939119	-4.15169716	2.81272661E-02	41
-2.93508887	-3.58501554	-9.35782939E-02	42
-0.408371031	-2.41236591	0.202707157	49
1.26204050	-2.48952603	0.191812649	50
3.67732048	-2.04206538	0.216945872	54
6.11606789	-2.20376420	6.71103131E-03	58
-6.05500841	-0.243756354	-4.88108583E-02	62
4.44241810	-1.26558888	0.231653884	65
-0.496863127	-0.626968205	6.99432893E-03	66
6.06402349	-0.412107378	-6.85489848E-02	73
-1.77124488	1.20534360	-4.25926708E-02	77
3.84134483	0.787184238	3.27881090E-02	78
-3.53733873	3.07216668	0.185950845	87
-1.87081778	2.98126507	0.193598241	89
0.666086435	4.15482569	-7.42995217E-02	95
-1.41841447	4.71670485	6.14446849E-02	96

Cluster Compound II-2

Br	-1.89564681	2.60405970	-8.95667151E-02
O	-3.70130730	0.194082052	1.50995874
Br	-3.85271263	-1.86141884	-0.916693509
C	0.321693003	-0.329891860	1.29794395
O	-0.721082926	-0.472576201	1.76223183
N	1.33543062	-1.24621928	1.46502388
H	1.06560385	-2.03978992	1.97733307
C	2.49638295	-1.18302405	0.842070997
N	3.35310841	-2.14619827	1.02487695
H	3.12831330	-2.93814421	1.55228782
H	4.19972277	-2.14242244	0.535713077
C	2.73758435	-8.94172341E-02	3.84086184E-02
Br	4.35248423	9.00889561E-02	-0.852792561
C	1.74637949	0.823687851	-0.120422043
H	1.85836148	1.65452957	-0.761276841
N	0.621035099	0.726042867	0.490139961
H	-0.180853799	1.37374723	0.298062712

H	-3.75056958	-0.339111090	0.739301741
H	-3.06507444	0.850096285	1.33960950

Coordinates and Index of Bond-Critical Points

-3.77530694	-0.817218423	0.225815937	24
4.04877186	-1.24742174	9.85253155E-02	28
-2.14464688	-8.47525075E-02	1.61535680	31
3.48664594	-9.22151003E-03	-0.373508722	37
-2.68623137	1.42987263	0.832833648	39
-0.690394700	1.74575448	0.178416371	42

Cluster Compound III-1

Br	-11.6176443	-1.48835874	-1.17361534
C	-9.80480671	-1.56494701	-0.852052569
C	-9.07887840	-0.386846751	-0.666148484
N	-9.55385399	0.797546446	-0.782762408
H	-10.4731855	0.980765939	-0.981208146
H	-8.97403049	1.55464292	-0.704574347
N	-7.79533958	-0.538104236	-0.334480911
H	-7.29195070	0.288860023	-0.194215387
C	-7.12705517	-1.70964527	-0.229024068
O	-5.99065495	-1.76435959	2.45438330E-02
N	-7.88001156	-2.79618263	-0.465776742
H	-7.44522476	-3.66857123	-0.521624088
C	-9.17261791	-2.73069715	-0.754538476
H	-9.69514084	-3.58842564	-0.906344354
O	-3.97182202	0.142306492	0.567910194
H	-4.53324795	0.897747040	0.464387268
H	-4.58893442	-0.498787165	0.324762404
Br	-2.63332462	2.91035557	0.582886875
C	-2.29489923	4.71720266	0.624777615
C	-3.25814843	5.58058119	0.405611575
H	-4.20170975	5.24551868	0.243181214
N	-3.01468444	6.89455318	0.393546492
H	-3.74714828	7.50316668	0.213279918
C	-1.78251231	7.41899967	0.590547740
O	-1.55947065	8.56004524	0.569683909
N	-0.816731870	6.50046539	0.842965901
H	7.43105561E-02	6.85021496	0.989787698
C	-0.972149968	5.19224691	0.876650393
N	2.80696452E-02	4.44673777	1.12632394
H	0.888132811	4.82191753	1.31784689

H	-6.73955306E-02	3.49874449	1.21307015
N	2.60284281	7.21016788	1.69943357
O	1.60998034	7.83061314	1.40048158
O	2.58250952	5.99427509	1.71551764
O	3.60079837	7.79459476	1.97892892
N	2.68885970	7.49464369	-1.48241460
O	2.71439791	8.69123840	-1.47406995
O	1.67262077	6.89818621	-1.75663888
O	3.68651843	6.86364794	-1.21620679
C	2.20564866	2.79853797	-1.34789765
O	1.04161596	2.83620811	-1.49635947
N	2.97681856	3.89610934	-1.26788080
H	2.58717012	4.79095602	-1.31870282
C	4.28671074	3.84507704	-1.09720302
H	4.81858587	4.70867586	-1.04723191
C	4.91328955	2.68760371	-0.989634395
Br	6.74511719	2.61732793	-0.804183364
C	4.17227554	1.50589418	-1.04923356
N	4.68292618	0.333873630	-0.941525519
H	4.14164639	-0.461072743	-1.03820133
H	5.59469986	8.46756175E-02	-0.770171642
N	2.88333154	1.62526059	-1.24922502
H	2.36488152	0.807209969	-1.23057222
N	1.50507081	1.39909303	1.70971215
O	0.306542993	1.53886139	1.53838682
O	2.22901702	2.33939409	1.67450345
O	1.92423415	0.285012960	1.90362084
H	-0.539708912	1.41801322	-2.00754976
H	-0.728619933	0.247056812	-2.76696610
O	-1.16643417	0.903872967	-2.29077530
N	1.68874907	-1.88647556	-1.31595731
O	1.20469201	-0.790581405	-1.24959743
O	1.00876975	-2.87382913	-1.30300689
O	2.89345980	-1.97997940	-1.38099062
O	4.17207527	-1.28073597	2.44441462
H	3.60806704	-0.521762609	2.34041333
H	3.55550861	-1.92125070	2.20148015
C	4.27654839	-5.56620264	-0.901003659
N	3.26222563	-4.81967497	-1.09378338
H	3.30675197	-3.85767961	-1.15324306
H	2.38129759	-5.17427826	-1.22809100
N	4.10202026	-6.88356829	-0.914425731

H	3.25716686	-7.28961611	-1.16366506
C	5.07630396	-7.81707525	-0.742148161
O	4.85468483	-8.95746708	-0.764178395
N	6.29605484	-7.29016638	-0.533868015
H	7.04771042	-7.93187904	-0.369205087
C	6.54404354	-5.98718405	-0.502988040
H	7.49419832	-5.66495132	-0.348659664
C	5.58138180	-5.10349035	-0.671180665
Br	5.91594172	-3.29838037	-0.616418123
C	0.975514650	-3.14704037	1.63569057
O	2.11548877	-3.20195818	1.89005637
N	0.312059671	-1.98388338	1.53096473
H	0.817311704	-1.15392888	1.67173624
C	-0.955564678	-1.83446836	1.20340657
N	-1.42789471	-0.656634390	1.08740795
H	-2.34668064	-0.473518103	0.889073133
H	-0.844684482	0.104869224	1.16603327
C	-1.67962515	-3.00946832	1.01799679
Br	-3.49174500	-2.93286228	0.696534157
C	-1.05930281	-4.17646551	1.11281633
H	-1.58156180	-5.03363848	0.961096048
N	0.219448105	-4.23800087	1.39799154
H	0.653763115	-5.10951233	1.34222078
N	0.921222866	-7.88190413	1.43237174
O	-7.76122063E-02	-7.25771523	1.13159895
O	0.899777353	-9.07929993	1.45310581
O	1.93390548	-7.28979397	1.71066034

Coordinates and Index of Bond-Critical Points

2.99356222	-7.09523106	0.413861096	105
0.343261600	-5.90362072	1.26408029	109
2.64589143	-4.02742434	0.432842880	124
-2.51534605	-2.97283530	0.868673384	126
5.73852015	-4.24166489	-0.646581769	129
-4.82732630	-2.26287961	0.319342792	132
2.99500775	-2.37638950	2.08592105	135
0.767053902	-2.59871531	0.155524805	136
-10.6735935	-1.52614200	-1.00755179	137
3.14512610	-3.19002819	-1.23901117	139
4.17872286	-2.77396274	-1.02928293	144
-3.89808345	-1.27480793	0.573586822	147
-5.13285255	-0.943257391	0.214731067	154

0.780386686	-1.47452796	9.18099657E-02	155
1.18994546	-0.631114542	1.74964964	160
-2.92532659	-0.234132558	0.775062799	164
2.98914742	-0.208736017	2.17119980	169
3.65287471	-0.995674729	-1.16729331	170
1.51436591	-0.309978813	0.344609082	171
-0.407216221	0.596935332	1.31152046	173
1.94849521E-02	-6.72758818E-02	-1.88310528	174
1.96617532	0.207975507	-1.24120688	179
-3.53649950	1.52732110	0.538044214	180
-1.83321035	1.81162524	-0.999548614	186
-0.948803067	2.31816220	1.10431278	188
2.51494670	2.03195024	0.220248550	190
0.763563037	2.21302438	4.14355546E-02	192
8.54379609E-02	2.78674173	1.33498776	194
8.49811733E-02	1.93555069	-1.81657791	195
-2.45220518	3.87256074	0.607450128	200
5.76848745	2.65119839	-0.902300775	202
0.548269510	3.66776490	-0.225037158	203
1.47963345	5.29088497	1.45576549	211
2.27981234	5.57309914	-1.47959399	218
3.04202628	5.55247927	-1.28420651	219
4.18926764	5.49491882	-1.14680970	220
0.623432934	7.17509699	1.14199114	226
3.11847019	6.94261408	0.295778990	228
2.17089176	7.81910086	-8.93315151E-02	235
4.93686628	-2.22471404	1.03961027	242
-1.27835548	9.41404477E-02	-0.638587892	245
2.72798920	5.00163460	0.222463936	252
0.523386538	6.74674797	-0.403200507	254
4.38898802	-0.455112487	0.794640541	257

Cluster Compound III-2

Br	-1.90713441	-1.23367798	2.66686510E-02
O	0.588055372	-2.74199367	0.155557394
C	2.90393710	0.295360744	4.01597004E-03
N	-0.170829505	2.94403601	3.12834121E-02
C	-3.68392706	-0.760655522	-8.34828708E-03
C	-4.67201328	-1.62949097	-7.51371915E-03
H	-4.49999285	-2.66876388	6.63131522E-03
N	-5.94700098	-1.24774897	-1.77161396E-02
H	-6.66985130	-1.91117918	-8.55355244E-03

C	-6.34119272	4.49307859E-02	-1.87130459E-02
O	-7.44666100	0.392782599	-1.41857080E-02
N	-5.29583693	0.928487718	-3.10671069E-02
H	-5.57887554	1.87098265	-3.81438956E-02
C	-4.00392246	0.625194609	-3.77870537E-02
N	-3.13541508	1.55050898	-6.83505684E-02
H	-2.14575911	1.40378439	-9.94937271E-02
H	-3.37940359	2.50321746	-7.39388093E-02
O	-0.350654095	1.75185537	-0.229227155
O	0.948681951	3.32701683	0.193538010
O	-1.09296978	3.67395401	0.130185887
N	1.70702553	-0.131878093	7.41789863E-03
H	1.49496603	-1.09486914	3.95813733E-02
H	0.951864064	0.516987026	-2.88495421E-02
N	3.12283635	1.60014939	-5.22662362E-04
H	2.33925700	2.22714877	2.34581511E-02
C	4.33678293	2.21766043	-2.82834582E-02
O	4.46931744	3.36894250	-5.30146770E-02
N	5.38293409	1.35156846	-2.31438987E-02
H	6.27394152	1.76182675	-3.70899066E-02
C	5.23809671	3.64262611E-02	-1.82412416E-02
H	6.12588501	-0.530881226	-2.85949185E-02
C	4.05035067	-0.538274467	-6.80164155E-03
Br	3.92039371	-2.38092780	-2.18129698E-02
H	-8.86095241E-02	-3.05969501	-0.398321450
H	1.17167711	-3.40059161	0.455264419

Coordinates and Index of Bond-Critical Points

2.09506702	-2.54991913	2.04826966E-02	40
-0.638267100	-2.13934636	0.107655957	41
1.15560067	-1.66774869	6.62660077E-02	45
-2.83790660	-0.984946370	6.02004817E-03	46
3.98389482	-1.40293515	-1.33443251E-02	48
0.530360520	0.967909455	-9.61711109E-02	63
-1.53908622	1.54819226	-0.146144077	68
1.84364808	2.56936240	7.89199024E-02	76

Cluster Compound III-3

C	-5.76121187	0.321752846	-1.01917779
N	-4.48369217	-4.73947488E-02	-0.957294643
C	-4.09052753	-1.33645868	-0.964579284
N	-5.10266447	-2.22149706	-1.01523304

C	-6.40603304	-1.93299687	-1.03136671
C	-6.73012447	-0.578878820	-1.06777740
O	-2.94693398	-1.66995513	-0.908774853
N	-7.25502062	-2.89472270	-1.02021039
Br	-8.48802853	-5.78886271E-02	-1.23482072
O	-1.59103763	-4.15300512	-0.860610843
N	1.26638114	-4.19584703	-0.690777838
C	2.11536860	-3.23412108	-0.701934040
N	3.41873741	-3.52262163	-0.685800493
C	4.43087482	-2.63758302	-0.635146618
N	4.03770971	-1.34851897	-0.627862096
C	2.76019073	-0.979371488	-0.689745307
C	1.79127741	-1.88000298	-0.738344908
O	5.57446766	-2.97107935	-0.579342306
Br	3.33744213E-02	-1.35901201	-0.905388474
O	4.23517895	-6.18294382	-0.571542263
N	3.43922210	-7.08968496	-0.472198904
O	3.79105091	-8.21505356	-0.224229515
O	2.24917436	-6.83179951	-0.621697545
O	6.93036461	-5.45412970	-0.531179249
O	4.52724171	1.46722662	-0.544711411
N	5.72331858	1.71860230	-0.583843291
O	6.52851057	0.811126232	-0.572572827
O	6.10655546	2.87879157	-0.634928465
N	2.69241476	3.46599746	-0.670368135
C	1.45454943	2.92476153	-0.663369834
N	0.462361693	3.81224847	-0.807962835
C	0.670613110	5.12775469	-0.910631955
C	1.89330983	5.62662792	-0.884970725
C	2.98438191	4.74627972	-0.770493686
O	1.27766991	1.75255299	-0.558693230
N	4.20762396	5.11757660	-0.762217343
Br	2.16195536	7.44160366	-0.970693648
C	0.651123822	1.91505826	2.34473634
N	1.64059901	1.01912308	2.49087143
C	1.44786704	-0.291651279	2.56084585
C	0.238195673	-0.808441997	2.48053217
C	-0.850747705	5.21539785E-02	2.33397412
N	-0.581399024	1.35482621	2.26680875
O	0.819773853	3.08299065	2.27626920
N	-2.06975317	-0.319225639	2.25985122
Br	-3.36221941E-02	-2.62053847	2.56336570

O	-2.42191577	3.32938218	2.17676973
N	-3.60384321	3.06885743	2.18286610
O	-4.40594530	3.97631717	2.16154408
O	-3.97567582	1.90488029	2.21163964
H	1.57369423	-5.11398745	-0.596029639
H	0.303998560	-4.06216526	-0.739244878
H	4.70302105	-0.654868245	-0.477494061
H	3.64229679	-4.46930647	-0.671313941
H	2.53503942	-3.29937898E-02	-0.698480546
H	6.49350357	-4.58708906	-0.469594777
H	6.12477016	-5.99843121	-0.565156698
H	-2.36132121	-1.24260211	2.35345936
H	-2.80027866	0.311465144	2.13753009
H	2.54948330	1.36464417	2.52163982
H	-1.28281951	2.02892351	2.26533937
H	2.21346569	-0.880843639	2.67535067
H	4.45428228	6.04954767	-0.892560840
H	4.92910099	4.46697760	-0.711291790
H	-0.452275068	3.48090792	-0.815227091
H	3.43171883	2.84185243	-0.569016874
H	-8.94358382E-02	5.72744799	-1.00598955
H	-6.94770718	-3.81286359	-0.925463140
H	-8.21740341	-2.76104140	-1.06867838
H	-3.81838179	0.646256089	-0.806926668
H	-4.87910509	-3.16818142	-1.00074685
H	-5.98636341	1.26813066	-1.02791321
H	-2.02789855	-3.28596354	-0.799026787
H	-2.39663196	-4.69730663	-0.894588351

Coordinates and Index of Bond-Critical Points

1.85094094	-5.68968391	-0.612151325	78
5.43819571	-6.06300735	-0.557226598	79
3.81728721	-5.06309271	-0.635880709	84
-0.380480111	-4.11763191	-0.780976117	87
0.716580212	-3.36807394	0.817997217	91
6.12393093	-4.03249979	-0.519930840	95
-2.39779687	-2.73837113	-0.848846495	100
-0.974146187	-2.82017803	-0.865637302	102
0.106771834	-1.66858554	2.51909781	104
-4.56819460E-02	-1.94425333	0.857236028	106
-1.57221961	-1.56994784	-0.883469343	114
-2.48353314	-0.976784289	0.615017354	116

0.975783885	-1.63907051	-0.813668132	119
0.122705847	-1.05104434	0.912467122	120
-7.57685232	-0.330561250	-1.14674067	126
-3.16974163	0.918113053	2.17138505	137
0.679292500	0.372519106	-0.720070601	144
4.56158400	0.137828469	-0.520088792	145
3.55663729	0.377379775	-0.626103401	148
2.05879235	0.675202191	-0.637535393	151
-1.68602383	2.44409490	2.23062134	153
0.999631763	1.94857645	0.917857289	156
3.81346822	2.38832593	-0.566586256	162
5.29430866	3.86363673	-0.682973385	170
2.02397418	6.49003839	-0.926644564	180
-1.04408562	3.44271636	0.671267152	187
2.02397418	6.49003839	-0.926644564	180
-1.04408562	3.44271636	0.671267152	187

Cluster Compound IV-1

Br	6.62897205	-2.88695860	-0.611891031
C	5.24460936	-1.64479578	-0.608255208
C	5.50853634	-0.314033806	-0.324669093
N	6.69311619	0.146970153	-8.50696564E-02
H	7.42610550	-0.520053864	-9.57088917E-02
H	6.78290606	1.08513987	0.284045994
N	4.49082899	0.497690469	-0.283801347
H	4.64415455	1.47487855	-0.113854647
C	3.18795538	0.150638431	-0.473660618
O	2.32425928	0.935436904	-0.406934232
N	2.99923992	-1.15973639	-0.715444505
H	2.05065513	-1.49633300	-0.711383164
C	3.96928596	-2.02493691	-0.778783619
H	3.74096751	-3.05592442	-0.970015705
S	5.64581490	3.81989551	1.32795560
O	6.46163082	2.64507914	1.61009228
O	6.47010708	4.91706419	0.905548453
O	4.91643620	4.22034216	2.50284743
O	4.67492676	3.44023991	0.253077537
O	2.28045344	4.22674561	0.593769848
H	3.22801638	3.85529280	0.391410172
H	1.80289555	4.18007088	-0.235384822
H	1.84958661	3.71974707	1.27288544
S	0.568317056	-3.95458055	-0.436469972

O	1.11996710	-4.81253672	0.592118979
O	1.46040201	-3.97398114	-1.61279762
O	0.456275403	-2.55905843	4.74849455E-02
O	-0.722548127	-4.47433853	-0.777911007
N	-2.35699844	-1.97640347	-1.93839148E-02
H	-1.36671352	-1.80679083	2.21981248E-03
H	-2.79711509	-2.84684825	0.283486873
C	-3.09110951	-0.951885641	-0.198564649
C	-2.57349563	0.377013296	-0.389133394
Br	-0.742622793	0.676363230	-0.333822548
C	-3.44289398	1.37691939	-0.503814459
H	-3.08022761	2.37146020	-0.616252065
N	-4.75259256	1.16259491	-0.481217235
H	-5.37286186	1.95682693	-0.433811903
C	-5.30679941	-6.59412071E-02	-0.335275888
O	-6.45364952	-0.265128821	-0.306377888
N	-4.40920305	-1.05976570	-0.198783770
H	-4.81377935	-1.98705053	-0.100083686
S	-5.25942469	-4.59763241	1.15192032
O	-3.93893385	-4.06590557	1.50119209
O	-5.83969831	-3.69873571	0.133364305
O	-6.12617254	-4.61645937	2.32120228
O	-5.14543486	-5.94835234	0.640441179
S	-5.30690193	4.79990625	4.41380180E-02
O	-4.50744724	4.41347265	-1.16530216
O	-4.40764952	5.14962769	1.11223364
O	-6.11508083	5.96367884	-0.235364184
O	-6.13560057	3.68793154	0.419059187
H	-3.70310950	5.80383682	-3.48597980
H	-3.86292100	5.20725107	-2.04024577
H	-2.41536450	5.49926424	-2.90155196
O	-3.28819013	5.83379650	-2.71170259

Coordinates and Index of Bond-Critical Points

-3.23848081	-3.27199388	0.710739374	63
-1.54415762	-3.15715384	-0.416905671	67
-5.16601801	-2.59836698	-3.82875255E-03	68
2.82079577	-3.34243965	-1.19077456	71
-0.718380630	-2.11046791	9.84741841E-03	73
1.49400258	-1.89014626	-0.435766101	77
5.87873411	-2.20885205	-0.610423386	78
7.03746271	-1.33804142	-0.287295878	86

-1.72955406	0.514786422	-0.364541620	94
0.872334540	0.759454072	-0.399822086	101
-5.62505579	2.59992385	-0.113833033	107
6.63798952	1.66029263	0.754767179	109
4.66335154	2.17701888	2.94313729E-02	111
2.38672304	2.58964634	9.08633545E-02	112
-3.70684195	3.15350628	-0.824309587	114
3.67791891	3.73484850	0.363328159	119
-4.05074453	4.99369335	-1.78358412	128
-0.316271394	4.79772425	-1.53419995	133

Cluster Compound IV-2

S	-2.70754790	-0.565019131	-0.768166423
O	-3.20100403	2.80917811	-0.336224437
O	-3.17698908	4.44951765E-02	2.71794605
O	-2.28851962	-3.47811031	1.03979254
N	0.567170560	0.951425612	-0.309700072
C	1.46596885	-1.15760369E-02	-0.382562786
C	2.76552129	0.308029652	6.40608519E-02
C	2.98391294	1.54624140	0.473495156
N	2.02229881	2.47102070	0.498785108
C	0.758686304	2.21915054	0.123951152
N	1.13511872	-1.14924884	-0.861215532
Br	4.10201120	-0.959945738	3.72058526E-02
H	0.187633857	-1.36509836	-1.03699803
H	1.79706395	-1.87342334	-0.883583367
H	-0.350783259	0.784319222	-0.664342940
O	-0.123892896	2.99023128	0.171477407
H	2.21918273	3.38418531	0.806393385
H	3.93445921	1.86101162	0.803047001
O	-2.05426621	0.660350800	-1.19884789
O	-1.66136730	-1.46766579	-0.277498960
O	-3.40228939	-1.14889586	-1.81923652
O	-3.68851638	-0.241919905	0.336714447
H	-1.99183977	-2.75631332	0.583407521
H	-2.19884920	-3.35790467	1.97286081
H	-1.76233447	-4.20772409	0.734224379
H	-3.40525937	-0.150379390	1.24452305
H	-3.47375178	-0.663435578	3.19550610
H	-3.65336347	0.783091486	3.04014874
H	-3.10562444	2.92387176	0.595760047
H	-2.85921359	3.55575299	-0.815948844

H	-2.72883892	2.09569049	-0.628274202

Coordinates and Index of Bond-Critical Points

-1.91203237	-2.34265900	0.310444355	35
-0.481268823	-1.36396098	-0.723370731	38
-3.34780049	-9.02065486E-02	1.69837987	41
3.40535212	-0.301746458	5.02056852E-02	49
-0.938665509	0.730343163	-0.829143047	52
-2.52798772	1.61696804	-0.799399793	56
-1.68171275	2.84162664	-9.64519829E-02	65

Cluster Compound IV-3

S	-2.74935985	-2.14235020	-1.96039467E-03
O	-3.23815918	1.39052236	-6.28701746E-02
S	-0.207088202	3.03434134	2.05092877E-02
O	0.286029130	-0.496189773	8.15378428E-02
S	3.66931200	-1.10413158	-3.07103246E-02
O	-3.89374399	-2.53859353	0.794799209
O	-2.37142348	-3.13163018	-0.967991889
O	-1.66330397	-1.86652231	0.925419807
O	-3.09070206	-0.884119511	-0.725389957
H	-4.08852625	1.70224488	-0.417983264
H	-3.15789247	0.434004337	-0.221080676
H	-2.49874234	1.88536441	-0.456309289
O	-1.33301461	2.73667789	-0.930820286
O	-0.648327947	4.06056643	0.943693399
O	4.55788411E-02	1.82013798	0.776566863
O	0.936122179	3.44198298	-0.732129931
H	-0.437071800	-1.00846016	0.473945588
H	0.237742171	0.452276647	0.338688523
H	1.17216265	-0.848958790	0.401440054
O	2.49860263	-1.50949156	0.784445941
O	4.04237652	-2.16565490	-0.957440734
O	4.78150702	-0.829158306	0.870705545
O	3.31927204	6.86947852E-02	-0.791040301

Coordinates and Index of Bond-Critical Points

-0.832950890	-1.27712965	0.593266904	28
1.58341277	-1.04712343	0.506185114	30
-3.13747907	4.30503562E-02	-0.358323634	35
0.172791347	0.869160950	0.455531329	40

-2.13898706	2.14656091	-0.581980884	43
2.03117681	1.67171800	-0.642620444	44

Cluster Compound V-1

N	3.77835560	-2.39193230E-03	-1.24532783
H	3.79746437	-1.00666797	-1.35495007
C	4.88628054	0.676832795	-1.05143166
H	5.80786276	0.167136118	-1.03933227
C	4.86978006	1.99978268	-0.870171726
Br	6.43465996	2.97688818	-0.658746064
C	3.63856673	2.64164567	-0.797250628
N	3.50318694	3.89570045	-0.525185227
H	4.29406023	4.45405531	-0.382708699
H	2.58724713	4.29811144	-0.459590852
N	2.57271433	1.91557479	-0.983205795
H	1.66468978	2.31107783	-0.949699163
C	2.56418610	0.576526046	-1.27130032
O	1.58173871	-9.87687963E-04	-1.51753676
P	4.62537766	-1.37382019	2.13728237
O	5.03371334	-1.62241781	3.59735012
H	4.64807463	-1.00758088	4.18468761
O	3.87866330	-0.123736389	1.99789619
O	5.97934628	-1.22876239	1.34641981
H	6.54608917	-1.96572399	1.44511199
O	3.90806723	-2.54338026	1.64540124
P	4.30863762	-3.98217583	-1.51885736
O	4.71696949	-4.23077726	-5.87884113E-02
H	4.35592842	-3.60017443	0.542044759
O	3.55598521	-5.13074064	-1.99600637
O	5.67870045	-3.88321137	-2.32739186
H	6.22161007	-4.63326025	-2.22567010
O	3.59309316	-2.70084333	-1.66631985
N	1.29986727	-2.36289263	1.08975935
H	2.18771124	-2.38141179	1.24151802
C	0.771950185	-3.58204317	0.763902366
O	1.43918645	-4.51618910	0.636089981
N	-0.584028780	-3.59838510	0.607039750
H	-0.994395435	-4.47791815	0.470319629
C	-1.33083379	-2.49704099	0.671247542
H	-2.38787651	-2.57432413	0.542539060
C	-0.793274879	-1.31331706	0.889400482
Br	-1.81614077	0.232655898	0.917505443

C	0.615466952	-1.24164295	1.15285587
N	1.21764529	-0.166564494	1.47341347
H	2.19940782	-0.183018133	1.67327130
H	0.720282078	0.678751945	1.52501285
C	-5.63083601	-0.850326061	-0.145074591
H	-4.68717909	-0.781332672	-3.01724430E-02
C	-6.18374825	-2.06783772	-0.369465411
Br	-5.16183043	-3.61239648	-0.397560567
C	-7.54861736	-2.13685846	-0.626190603
N	-8.16225910	-3.23238707	-0.952879071
H	-7.66536093	-4.07691526	-1.00445676
H	-9.12308121	-3.21627545	-1.14848483
N	-8.23404026	-1.01394141	-0.562979162
H	-9.20375443	-0.993707538	-0.728726983
C	-7.70602131	0.205452085	-0.237023816
O	-8.37423515	1.14095783	-0.109008111
N	-6.36774969	0.221580431	-8.21982622E-02
H	-5.94920731	1.11864352	5.72703257E-02
P	-4.26596308	3.60992718	0.376650721
O	-3.23888254	2.94868398	1.31758475
H	-2.40392041	3.37936020	1.31787252
O	-3.57415247	3.62173676	-1.05866599
H	-4.09868813	3.20813704	-1.71003437
O	-4.48424244	4.97268915	0.797973990
O	-5.49327278	2.78250694	0.377259970
P	-0.225585684	4.29166794	-0.355489075
O	-0.892104626	4.28148603	1.02870393
H	-0.366518438	4.69628572	1.68051052
O	-1.26336646	4.95874214	-1.30608451
H	-2.09718680	4.52865601	-1.30568063
O	0.988129079	5.10992098	-0.357263774
O	-3.03636328E-03	2.90342784	-0.780420661

Coordinates and Index of Bond-Critical Points

-6.78471899	-3.71345091	-0.753341734	73
2.61142063	-4.57105207	-0.544258893	75
3.03587961	-4.32102203	0.206599355	76
-3.41247439	-2.95688748	0.187678829	82
-5.70703506	-2.79177976	-0.384347349	83
4.46753263	-4.06277561	-0.939254045	85
4.22270823	-3.23723340	0.910540938	90
4.85387325	-3.96337771	-1.82282615	92
2.79491019	-2.42002511	1.39468253	97

2.46520829	-2.55785465	-0.262116790	99
4.77969551	-1.45478177	2.71817541	107
5.16576147	-1.33937430	1.83990514	112
-1.27897954	-0.577981353	0.904012978	118
-3.65299916	-0.438007057	0.316388488	119
3.74691200	-1.59390879	-1.45483887	121
2.77648211	-0.180632651	1.78486085	125
1.43234432	-9.27975029E-02	-4.93629500E-02	127
5.68646526	-0.362014949	3.33725475E-02	132
3.91139269	-4.74058352E-02	0.385143965	134
-5.76988983	1.68586183	0.161628157	135
-2.56212592	1.62184823	1.12190485	136
-0.833455086	1.67071319	4.10988107E-02	141
-3.84300780	3.35766459	0.733856380	148
1.09040058	2.53468180	-0.881204724	150
-1.87282050	3.70022392	1.21021378	153
-4.00788498	3.59943390	-0.192111105	154
5.58618736	2.44980955	-0.771681786	156
-0.477550060	4.29741287	0.196988583	163
-2.61185265	4.20546722	-1.21595550	164
4.98098326	3.75318480	-0.522614121	166
-0.653515697	4.53903055	-0.716800451	167
2.01991343	4.56472635	-0.423574537	172

Cluster Compound V-2

N	1.86166561	-0.432216257	-2.94257067E-02
O	-0.643727541	-1.61082757	8.39059576E-02
C	-1.74818695	-1.23926687	4.79810014E-02
P	0.455890447	2.93280745	0.135171384
C	3.11316299	-0.194323957	-6.55928403E-02
C	4.08001184	-1.23408377	-3.90636772E-02
Br	3.55606771	-3.00363469	5.09477854E-02
C	5.35332680	-0.903336346	-8.42820629E-02
H	6.11884117	-1.62712657	-6.98782578E-02
N	5.73833847	0.362716019	-0.150145218
H	6.68926573	0.600310385	-0.185487539
C	4.87273645	1.41084218	-0.170840204
O	5.23243523	2.51308250	-0.222177193
N	3.56117821	1.04573166	-0.125767365
H	2.91473150	1.83388901	-0.121174335
O	3.20394598E-02	1.53091836	-4.16470841E-02
O	1.88943923	3.13748813	-3.10865659E-02

O	2.69078966E-02	3.48664927	1.53651929
H	0.711449862	3.99846625	1.91276526
O	-0.313179255	3.81235838	-0.909801900
H	0.218020901	4.51643944	-1.21660054
N	-2.10111380	5.06567284E-02	-7.53398761E-02
H	-1.32482541	0.722285748	-8.91114250E-02
C	-3.34783053	0.446318865	-0.120363101
H	-3.49774647	1.48632276	-0.212582991
C	-4.37755871	-0.403531224	-5.52928597E-02
Br	-6.12647343	0.179997429	-0.126930848
C	-4.08467579	-1.75615025	7.40215108E-02
N	-4.98515224	-2.67595744	0.150851488
H	-5.93492746	-2.43323159	0.119156294
H	-4.76263380	-3.62548876	0.244677797
N	-2.80735230	-2.10233498	0.119413435
H	-2.54943824	-3.04709482	0.207825676
H	1.18514645	0.304600269	-4.52941507E-02
H	1.52778161	-1.35050952	1.53266350E-02

Coordinates and Index of Bond-Critical Points

3.83038163	-2.06386185	3.22656403E-03	42
2.43866396	-1.78348088	1.83530319E-02	45
0.626572430	-1.29264402	3.49101461E-02	47
-5.20966482	-0.128968120	-8.90571401E-02	51
0.813763201	0.728225112	-3.91076282E-02	65
-0.894445896	0.991614580	-6.87539130E-02	66
2.55924964	2.24841356	-8.60324949E-02	74
0.299429357	3.15800095	0.685025752	75
0.165546760	3.28946018	-0.272607893	77

Cluster Compound VI-1

C	-2.60934186	1.22681642	-0.250621945
O	-3.32733440	2.12690115	-0.151454091
N	-1.27557206	1.36711740	-0.428121328
H	-0.934231222	2.31056881	-0.470262587
C	-0.467890352	0.356464922	-0.538174033
H	0.573208749	0.533850372	-0.659080207
C	-0.917301834	-0.904560864	-0.502687335
Br	0.222410008	-2.37322021	-0.615239024
C	-2.31031418	-1.12076521	-0.331019044
N	-2.84027028	-2.29469323	-0.263399422
H	-3.85212731	-2.33761191	-0.197456226

H	-2.25909305	-3.07452345	-0.442317784
N	-3.05474329	-5.74973933E-02	-0.216880426
H	-4.04305696	-0.155658051	-8.58371407E-02
O	-5.74495411	3.24757004	-1.02014601
H	-5.18382120	2.63640428	-0.571233630
H	-5.85943556	2.76871419	-1.82445908
O	-1.40043998	-5.44214582	-0.620842099
H	-1.60407495	-5.89942408	-1.42216003
H	-2.13986635	-5.72541809	-0.105155200
C	-6.28516960	-1.32804036	0.431466222
O	-5.83236027	-0.214632332	0.307814479
O	-5.69445992	-2.36291814	0.215858385
C	-7.72287846	-1.47511673	0.918367147
O	-8.33901882	-0.344242036	1.03916717
H	-9.25457764	-0.385416418	1.25614679
O	-8.16273212	-2.52570367	1.15137494
C	0.359423548	4.78130102	-0.565071404
O	0.477674544	5.96552277	-0.670643210
O	-0.663293958	4.15884972	-0.697889328
C	1.61306775	3.94649243	-0.216768399
O	1.52222490	2.78807068	-3.40101309E-03
O	2.69291782	4.64317703	-0.182342961
H	3.49556637	4.16801357	-5.73381037E-02
O	1.88654888	7.40204000	1.27676594
H	1.68212378	6.94292498	0.472240627
H	1.14956760	7.11971331	1.79070961
C	4.40018702	-1.08892393	0.402625233
Br	3.26212835	0.377618760	0.515006840
C	5.77750874	-0.875152528	0.232891783
N	6.30731440	0.298450142	0.165286899
H	5.72439289	1.08061802	0.344738841
H	7.29575968	0.340377241	0.100870751
N	6.54759073	-1.94259572	0.115720667
H	7.52669430	-1.84533417	-1.40962582E-02
C	6.10069609	-3.23124409	0.149580091
O	6.81806135	-4.13054180	5.04869446E-02
N	4.77338743	-3.37087393	0.326204926
H	4.43617058	-4.30293798	0.367814600
C	3.94832015	-2.33847713	0.438638657
H	2.90349865	-2.51650429	0.559964418

Coordinates and Index of Bond-Critical Points

-1.92269993	-3.96442366	-0.530094266	54
-0.772761166	-3.96224141	-0.615668952	55
-4.50190353	-2.31930876	-4.90295738E-02	60
-1.39145494	-2.63883209	-0.470574468	63
1.94264400	-2.40539455	0.133244470	71
-0.397354007	-1.57321525	-0.553573310	78
1.78612745	-1.02253067	-3.08083035E-02	84
-4.66558838	-0.195495605	5.04095368E-02	85
3.85079288	-0.378955752	0.458757937	91
1.56797910	0.404455900	-0.215051740	95
-5.47434855	1.44465017	-0.246753171	99
4.87742424	0.687517405	0.379157722	103
0.855333924	1.51178575	-0.365471005	106
2.41280770	1.65489566	0.199774697	107
-4.47212362	2.44227052	-0.431160718	109
-0.815730453	2.95956898	-0.548370183	113
1.24077582	6.56947899	6.96227178E-02	120

Cluster Compounds VI-2

C	-1.13629246	1.11922741	-0.467528224
N	-2.25841713	1.81310582	-0.300508201
C	-3.45999146	1.23143768	-0.131765664
N	-3.45261145	-0.129282326	-0.165890083
C	-2.36595583	-0.877743959	-0.340318531
C	-1.14038038	-0.209242195	-0.498421699
O	-4.46848822	1.85626400	1.97361149E-02
N	-2.47411299	-2.15938520	-0.334786683
Br	0.400320828	-1.19450331	-0.685630381
O	-5.83302212	-1.12562954	0.323686540
C	-5.88866138	-2.34873581	0.389342815
C	-7.18309355	-2.98704791	0.857667029
O	-8.12539291	-2.14345717	1.03573644
O	-4.97837877	-3.12712812	0.115618236
O	-7.24823713	-4.16083288	1.03357828
O	-2.63007951	4.44727898	-0.427357554
C	-1.85504186	5.38548040	-0.258449733
C	-0.416447341	5.00632334	3.98968123E-02
O	0.365839839	6.01344585	0.110705562
O	-2.14634514	6.57568693	-0.302528709
O	-0.111574873	3.86762404	0.192794695

O	-7.13586187	2.15033388	-0.788999796
O	2.79376435	-2.81811023	-0.279201329
C	3.09863710	-3.95681047	-0.126302600
C	4.53723097	-4.33596659	0.172043085
O	4.82853460	-5.52617359	0.216122031
O	5.31226921	-3.39776540	0.340951771
O	2.31634974	-4.96393204	-0.197112218
O	-0.201345146	-4.54384184	-0.823361754
N	4.94060659	-0.763592541	0.214102417
C	6.14218092	-0.181924507	4.53598760E-02
N	6.13480043	1.17879546	7.94843584E-02
C	5.04814434	1.92725706	0.253912807
C	3.82256985	1.25875664	0.412015498
C	3.81848192	-6.97137266E-02	0.381121576
O	7.15067768	-0.806750894	-0.106141903
N	5.15630150	3.20889831	0.248380959
Br	2.28186870	2.24401641	0.599224627
H	6.05615568	3.57354450	0.187589452
H	4.36526585	3.73230505	0.464502364
H	4.93321657	-1.73636639	0.206617147
H	7.00595474	1.59428191	-4.24233377E-02
H	2.97586727	-0.545151532	0.482906014
H	1.36843634	-4.77105618	-0.300355464
H	-0.813061535	-5.09821796	-0.308706701
H	-0.256945819	-5.01592636	-1.67215168
H	1.31375325	5.82056952	0.213949665
H	-6.36573458	1.72247684	-0.355954766
H	-7.09751797	1.68715847	-1.64363301
H	-9.00044250	-2.50919366	1.25235617
H	-3.37396741	-2.52403069	-0.273996919
H	-1.68307686	-2.68279099	-0.550907671
H	-2.25102711	2.78587961	-0.293022960
H	-4.32376575	-0.544768810	-4.39823978E-02
H	-0.293678820	1.59466600	-0.569312155

Coordinates and Index of Bond-Critical Points

0.856048226	-4.70703173	-0.483059347	60
-1.12837827	-3.39058018	-0.675579667	69
-3.92577648	-2.69937587	-0.133231059	71
-4.44180779E-02	-2.98404336	-0.759270012	74
-1.01357913	-1.99678171	-0.560855925	79
1.61740863	-2.09350681	-0.421524733	81

5.04240322	-2.29004407	0.251496047	82
-4.81276894	-0.752060354	7.76172653E-02	84
3.04069710	-1.48718083	0.144044712	85
-0.416620195	-0.672022402	-0.588582039	90
2.01981878	-0.705802560	5.30723818E-02	91
1.36026132	0.533955276	-4.40946296E-02	100
-5.65907860	1.77690053	-0.254295439	108
0.679980338	1.76762927	-0.134048998	112
3.08711576	1.73087180	0.503577769	113
-0.343165338	2.54440141	-0.229204997	118
-2.35974956	3.34378576	-0.338571936	120
1.07838368	3.15202785	0.338882983	121
3.69217658	3.08041883	0.479638845	123
